# A large-scale multi-ancestry mitochondrial variant association analysis for cardiometabolic traits

**DOI:** 10.1038/s41467-026-74939-4

**Published:** 2026-07-04

**Authors:** Jin J. Zhou, Aubrey Jensen, David C. Samuels, Kyriacos Markianos, Hua Zhou, Hinn Zhang, Marijana Vujkovic, Julie A. Lynch, Tia Dinatale, Jacob Joseph, Chunyu Liu, Adriana Hung, Yan V. Sun, Saiju Pyarajan, Philip S. Tsao, Kyong-Mi Chang, Todd Hulgan, Peter Reaven, VA Million Veteran Program, Jin J. Zhou, Aubrey Jensen, David C. Samuels, Kyriacos Markianos, Marijana Vujkovic, Julie A. Lynch, Tia Dinatale, Jacob Joseph, Adriana Hung, Yan V. Sun, Saiju Pyarajan, Philip S. Tsao, Kyong-Mi Chang, Todd Hulgan, Peter Reaven

**Affiliations:** 1 Department of Biostatistics, UCLA Fielding School of Public Health, Los Angeles, CA USA; 2 Phoenix VA Health Care System, Phoenix, AZ USA; 3 Department of Molecular Physiology and Biophysics, Vanderbilt University School of Medicine, Nashville, TN USA; 4 VA Boston Healthcare System, Boston, MA USA; 5 Department of Cognitive Sciences, UCI School of Social Science, Irvine, CA USA; 6 Corporal Michael J. Crescenz, VA Medical Center, Philadelphia, PA USA; 7 Department of Medicine, University of Pennsylvania Perelman School of Medicine, Philadelphia, PA USA; 8 VA Informatics and Computing Infrastructure (VINCI), Salt Lake City VA, Salt Lake City, UT USA; 9 Department of Internal Medicine, Epidemiology, University of Utah School of Medicine, Salt Lake City, UT USA; 10 Cardiology Section, VA Providence Healthcare System, Providence, RI USA; 11 Department of Medicine, Brown University, Warren Alpert School of Medicine, Providence, RI USA; 12 Massachusetts Veterans Epidemiology Research and Information Center, VA Boston Healthcare System, Boston, MA USA; 13 Department of Biostatistics, Boston University School of Public Health, Boston, MA USA; 14 Department of Medicine, Vanderbilt University School of Medicine, Nashville, TN USA; 15 Veterans Affairs Tennessee Valley Healthcare System, Nashville, TN USA; 16 Veterans Affairs Atlanta Healthcare System, Decatur, GA USA; 17 Department of Epidemiology and Global Health, Emory University Rollins School of Public Health, Atlanta, GA USA; 18 Department of Biomedical Informatics, Emory University School of Medicine, Atlanta, GA USA; 19 Department of Medicine, Stanford University School of Medicine, Stanford, CA USA; 20 VA Palo Alto Health Care System, Palo Alto, CA USA; 21 Department of Medicine, Division of Infectious Diseases, Vanderbilt University Medical Center, Nashville, TN USA; 22 College of Medicine, University of Arizona, Phoenix, AZ USA

**Keywords:** Genetics, Endocrinology

## Abstract

Studies linking mitochondrial DNA (mtDNA) with complex traits are often limited by small sample sizes or focused on specific phenotypes in clinically selected cohorts. Here, we use data from >600,000 participants in the Million Veteran Program (MVP) to perform a multi-ancestry analysis of mitochondrial DNA (mtDNA) variation and cardiometabolic phenotypes across European (EUR), African (AFR), Admixed American (AMR), and East Asian (EAS) populations. After validating 248 mtDNA loci, we identify 10 ancestry-stratified single-variant associations (8 EUR, 2 AFR) and 23 additional signals in sex- and type 2 diabetes (T2D)–stratified analyses. Four variants tagging haplogroup J, D-loop MT228G > A, *MT-ND3* MT10398A > G (p.Thr114Ala), *MT-ND5* MT13708G > A (p.Ala458Thr), and *MT-CYB* MT14798T > C (p.Phe18Leu), are associated with hypothyroidism in EUR and replicated in UK Biobank (UKBB) with concordant effects. In EUR females, *MT-RNR1* MT1555A > G increases the risk of carditis and heart failure phenotypes, supporting prior reports of maternally inherited cardiomyopathy. Gene-based rare-variant tests (minor allele frequency ≤2%) yield 26 associations (12 EUR, 10 AFR, 2 AMR, 2 EAS), including mitochondrial tRNA burdens linked to primary cardiomyopathy (females) and exophthalmos (males). Twenty-three of the 33 single-variant signals map to the endocrine/metabolic category, indicating significant enrichment (Fisher’s exact *P* = 0.003). These results define ancestry- and context-specific contributions of mtDNA to cardiometabolic disease, with a notable concentration in endocrine traits, and provide a framework for mtDNA analysis across diverse biobank cohorts.

## Abstract (web summary)

Here the authors analyse mitochondrial DNA in more than 600,000 U.S. veterans and link mitochondrial genetic variation to heart, hormone, and metabolic diseases, with risks that differ by ancestry, sex, and diabetes status.

## Introduction

The human mitochondrial genome, spanning 16,569 base pairs, primarily comprises components crucial for ATP generation through oxidative phosphorylation. It is maternally inherited as a single unit and does not undergo intermolecular recombination. Variations in the genetic material of mitochondria form geographic region-specific macro-haplogroups. These variations can directly alter mitochondrial function or co-occur with changes that influence mitochondrial metabolism. Consequently, mitochondria are reported to play an essential role in a variety of common cardiometabolic conditions, such as type 2 diabetes mellitus (T2D)^1,2^.

Previous studies linking mitochondrial DNA (mtDNA) with complex traits were often limited by small sample sizes or focused on specific phenotypes in clinically selected cohorts^3^. Recently, a few mtDNA association studies conducted in large-scale biobanks, coupled with deep phenotypic data, have provided an opportunity to address these issues and offer robust findings^4,5^. However, none have included Admixed American and African ancestries. Using the Million Veteran Program (MVP) with a uniquely designed genotyping platform, we report a mitochondrial association study of cardiometabolic traits to date across four ancestry groups: European (EUR), African (AFR), Admixed American (AMR), and East Asian (EAS). By exploring these variants, we aim to uncover potential mitochondrial genetic markers for various health conditions and contribute to a broader understanding of mitochondrial genetic diversity in health and disease. We also tested whether mitochondrial genetic variants could lead to different clinical outcomes between sexes and among distinct metabolic environments, such as T2D, as previously suggested^6,7^. This reflects our foundational efforts to create an atlas of mitochondrial genetic associations with diseases and endophenotypes in a genetically diverse cohort.

## Results

### Characteristics of study participants and mtDNA variants

The MVP study cohort included 434,891 EUR, 111,840 AFR, 54,258 AMR, and 10,381 EAS participants. Cohort generation steps are detailed in Supplementary Fig. 1. The participants’ mean age was 57 years, ranging from 20 to 100 years. There were 31,818 (7.3%), 15,577 (13.9%), 5,581 (10.3%), and 1,358 (13.1%) females and 165,696 (38.1%), 52,160 (46.6%), 21,951 (40.5%), and 3,575 (34.4%) T2D patients in each ancestry group (Table 1). In general, female veterans were younger and healthier than male veterans. Veterans with T2D were older (mean ages ranged from 59 to 67 across the four ancestry groups; Table 1, Bottom Panel). The number of cases (defined by PheCode) and their proportions across four ancestry groups are shown in Supplementary Data 1.

**Table 1:** MVP cohort characteristics by genetically inferred ancestry, sex, and diabetes status

‡ Mean (SD); § (N (%) *PheCode present; Individuals with Type 1 Diabetes (T1D) were excluded in the bottom panel of the subgroup analysis stratified by T2D status.

*MVP* Million Veteran Program, *EUR* European ancestry, *AFR* African ancestry, *AMR* Admixed American ancestry, *EAS* East Asian ancestry, *SBP* systolic blood pressure, *DBP* diastolic blood pressure, *HDL* high-density lipoprotein cholesterol, *LDL* low-density lipoprotein, *BMI* body mass index, *T2D* Type 2 Diabetes, *HbA1c* hemoglobin A1c, *SD* standard deviation.

Call rates were high for mtDNA variants. Among 248 mtDNA variants, 98% (243/248) of the markers passing QC had a call rate above 99% (Supplementary Fig. 2). Concordance between intentional duplicates confirmed the consistency of the genotyping assay: 98.43% of the 30,347 sample pairs had perfect concordance across all mitochondrial markers. 89.7% of the markers perfectly matched across all genotype pairs. Concordance between chip and WGS genotypes confirms the validity of genotype calling using an independent typing assay (Supplementary Fig. 3-4). Among the 1835 chip-WGS pairs, 99.5% had identical mtSNV calls. Among polymorphic markers, 95.9% calls were identical across all 1835 chip-WGS pairs (Supplementary Fig. 5-6). A further, in-depth discussion of mtDNA QC is available in the Supplementary Materials. We present a comparison between the MVP mitochondrial allele frequency distribution (across all ancestries) and that from the UK Biobank (UKBB), among others (Supplementary Fig. 7). In addition, the gnomAD database (v4.1) contained 217 out of 248 MVP mtSNVs (Supplementary Data 2). In EUR and AFR ancestries, the two databases showed highly concordant allele frequencies (Pearson’s correlation 0.935 and 0.925) (Supplementary Fig. 8). The concordances were slightly lower for the other two ancestries, i.e., 0.880 for AMR and 0.831 for EAS, with higher variations due to the smaller sample sizes and lower minor allele counts in these ancestries.

**Fig. 1:**
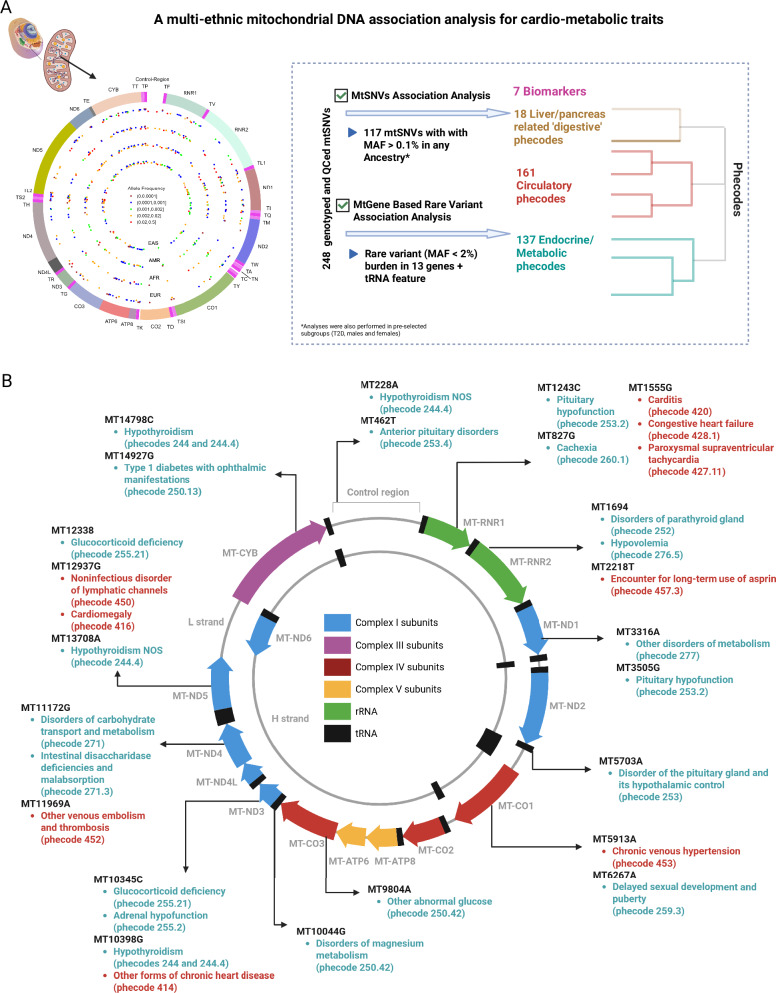
Schematic of methodology and the significant mtSNVs identified in the study. **A** Schematic of the methodology. Color coding represents the phenotype category. Pink: biomarker; Brown: digestive chapter PheCodes; red: circulatory chapter PheCodes; dark cyan: endocrine/metabolic chapter PheCodes; **B** Significant mtSNVs. MtSNVs (from Table 2, Figs. 2, 3) were annotated to their corresponding genes, and each bullet represents the traits that mtSNV was associated with. Stratified population annotations (i.e., ancestries, sex, and T2D) were not shown due to space limitations. Created in BioRender. Zhou, J. (2026) https://BioRender.com/3fw3yjm. Source data are provided as a Source Data file.

There were 98 (39.5%), 111 (44.8%), 117 (47.1%), and 124 (50.0%) genotyped mtDNA variants that did not vary in the EUR, AFR, AMR, and EAS ancestries, respectively. Of the remaining mtSNVs, 12 (4.8%), 24 (9.6%), 9 (3.6%), and 11 (4.4%) had minor allele frequencies (MAF, calculated within each analysis population) > 2% (Fig. 1A, Left Panel), and 49 (19.8%), 52 (21.0%), 75 (30.2%), 62 (25%) had MAF between 0.1% and 2% in the EUR, AFR, AMR, and EAS ancestries, respectively (Supplementary Data 3). There were no eligible genotyped variants in the *MT-CO2* and *MT-ND4L* genes. We also excluded seven multi-allelic mtSNVs and mtSNVs with population-specific call rates <98%.

### MtDNA variants and cardiometabolic traits

#### MtSNV association analysis in ancestry-stratified populations

In total, we investigated 31,122 unique mtSNV × trait pairs across all ancestries and subgroups, for a total of 232,825 association tests (Fig. 1A, Right Panel). Ten unique pairs (8 from EUR and 2 from AFR) were significant in the ancestry-stratified analysis. Six of the 8 associations in EUR were for hypothyroidism, and 8 of 10 associations were related to endocrine glands, specifically the thyroid, pituitary, or adrenal glands. All 10 associations reflected PheCodes in the “endocrine/metabolic” chapter (Table 2). The genomic inflation factor for each condition was reported (Supplementary Data 4).

**Table 2:** Significant mtSNV associations with cardiometabolic PheCodes identified across EUR, AFR, AMR, and EAS ancestries in MVP

*EUR* European ancestry, *AFR* African ancestry, *AMR* Admixed American ancestry, *EAS* East Asian ancestry, *BP* base pair, *REF* reference allele, *ALT* alternative allele, *Alt AF* alternative allele frequency, *OR* odds ratio, *CI* confidence interval, *Adj.* *P*-value p-value adjusted by Benjamini–Hochberg procedure, *NOS* Not Otherwise Specified, *NEC* Not Elsewhere Classified; Analyses were not conducted when the number of cases was less than 500 or the MAF was less than 0.1% in the testing population, and the corresponding OR and *p*-values were marked as “-“. Results with adjusted *p*-value < 0.05 were highlighted in bold font. The minor allele (calculated within each ancestry) was the alternative effect allele. All the minor alleles are consistent across ancestries except allele A of the MT10398 variant, which is the minor allele in AFR and EAS ancestries (highlighted in bold font). However, allele G is the minor allele in the EUR and AMR ancestries. All reported conditions were PheCodes from the endocrine/metabolic chapter. Firth logistic regression and two-sided *P* values based on Wald Z-score were reported.

In the EUR ancestry, non-coding variant MT228G > A, located within the *MT-D-loop*, significantly elevated the risk of “hypothyroidism” (PheCode 244.4) (Table 2) with an estimated OR (95% CI) of 1.08 (1.04-1.12). The estimated effects among AFR ancestry were comparable to those in EUR, although not statistically significant. Estimated effects in the AMR ancestry were more neutral. Among EAS ancestry, the estimated effect of MT228G > A was associated with reduced prevalence of hypothyroidism, despite wide confidence intervals, likely due to small sample sizes. Additionally, missense mutations MT10398A > G in *MT-ND3* (T114A), MT13708G > A in *MT-ND5* (A458T), and MT14798T > C in *MT-CYB* (F18L) were also linked to a significantly higher risk of hypothyroidism in EUR. Effect sizes for these variants in AFR, AMR, and EAS ancestries were either weakly increased risk or neutral. All four variants were common in the EUR ancestry, each with allele frequency exceeding 5%, while the minor allele frequencies in non-EUR ancestries only exceeded 5% for MT10398A > G. Extended PheWAS analyses showed that MT10398A > G was significantly associated with ptosis of the eyelid (PheCode 374.3) and hematemesis (PheCode 578.1). Both phenotypes have been described in primary mitochondrial disorders, with ptosis representing a hallmark feature of chronic progressive external ophthalmoplegia and hematemesis occurring in cases with severe gastrointestinal involvement in MELAS (Mitochondrial Encephalomyopathy, Lactic Acidosis, and Stroke-like episodes) and other mitochondrial diseases^8,9^ (Supplementary Fig. 9). MT13708G > A is one of the defining variants for haplogroup J, a relatively common haplotype across Europe (2-20%), and the Near East (19%)^10^. MT14798T > C defines subgroup J1c, which is most frequent in Europe^11^. In MVP, the prevalence of hypothyroidism (PheCode 244.4) was notably higher in EUR and AMR ancestry groups, 15.6% and 11.3%, respectively, vs. 7.5% and 7.3% in the AFR and EAS groups. The latter was similar to that reported in the US general population, i.e., around 6%^12^. All four variants, *MT-D-loop* MT228G > A, *MT-ND3* MT10398A > G, *MT-ND5* MT13708G > A, and *MT-CYB* MT14798T > C, replicated in UKBB with similar effects (OR 1.05–1.10) using PheCode 244.4 or the ICD-10 E03 first-occurrence field (Table 3).

**Table 3:** Replication of MVP EUR mtSNV associations in UKBB

*MVP* Million Veteran Program, *mtSNV* mitochondrial single nucleotide variant, *UKBB* UK Biobank, *EUR* European ancestry, *T2D* Type 2 Diabetes, *BP* base pair, *REF* reference allele, *ALT* alternative allele, *Alt AF* alternative allele frequency, *OR* odds ratio, *CI* confidence interval; Firth logistic regression and two-sided *P* values based on Wald Z-score were reported.

In individuals of EUR ancestry, we also identified a significant association between MT6267G > A (MAF = 0.14%), a missense mutation (A122T) in the *MT-CO1* gene, and the endocrine condition “delayed sexual development and puberty” (PheCode 259.3). The most similar PheCode (defined by code co-occurrence) to this PheCode, extracted by the KESER^13^ (Supplementary Data 5), was “pituitary hyperfunction” (PheCode 253.1); it was also the most similar PheCode to “anterior pituitary disorders” (PheCode 253.4). MT14927A > G (MAF = 0.10%), a missense mutation T61A in gene *MT-CYB*, was found to increase the risk of “type 1 diabetes with ophthalmic manifestations” (PheCode 250.13), OR (CI 95%) = 3.01 (1.87-4.87), *p*-value = 6.3e-6). The similar PheCode features to this condition include diabetic retinopathy and diabetes (Supplementary Data 5).

We identified two significant associations in the AFR ancestry group with MT462C > T and MT10345T > C (Table 2). 1,013 veterans (0.9% of the AFR cohort) were found to have “anterior pituitary disorders” (PheCode 253.4). Notably, this association with MT462C > T (MAF = 0.36%) persisted in both the T2D and male-only subgroups within the AFR cohort. Extended PheWAS also showed MT462C > T was associated with sicca syndrome (PheCode 709.2) in the AFR male-only subgroup (Supplementary Fig. 10). We did not analyze MT462C > T in EUR or EAS ancestries because call rates were lower in these ancestries. MT10345T > C (MAF = 0.11%), a missense mutation (I96T) in *MT-ND3*, was significantly associated with “glucocorticoid deficiency” (PheCode 255.21). This variant exhibited an MAF > 0.1% and >500 cases exclusively in the AFR and male-only AFR ancestry cohorts, preventing testing in other ancestral groups.

#### MtSNV sex-stratified analysis across 4 ancestry groups

**Fig. 2:**
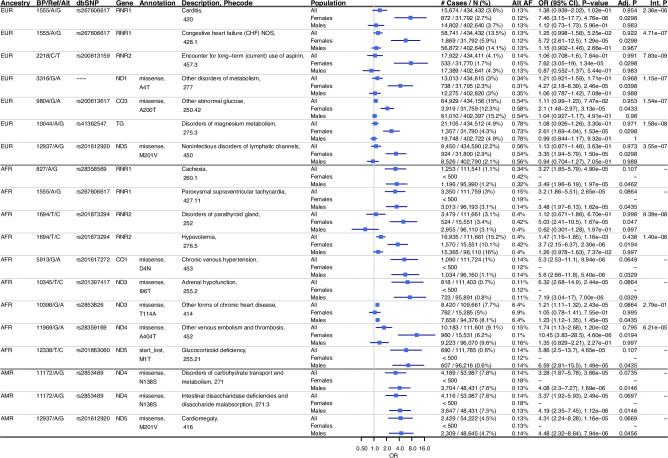
Significant mtSNV associations in sex-stratified analysis across MVP ancestry groups. Firth logistic regression and two-sided *P* values based on Wald Z-score were reported. Results from other groups were also included for comparison purposes. Note, for mtSNVs significantly associated with a phenotype in one subgroup, but not in the overall group for that ancestry, we analyzed and tested for statistical interactions with sex using logistic regression. Significant associations among subgroups that were also significant in the overall group are not shown. EUR European ancestry, AFR African American ancestry, BP mtSNV base pair, REF reference allele, ALT alternative allele, Alt AF alternative allele frequency, CI confidence interval;Adj. *P* *p*-value adjusted by Benjamini–Hochberg procedure, Int. *P* *p*-values for mtSNV × sex interaction effects. Interaction analyses (mtSNV × sex) were only conducted if the number of cases was > 500 and mtSNV allele frequencies were > 0.1% (refer to Methods for details), and the corresponding *p*-values were marked as “-”. These analyses included OR (95%CI) for subgroup effects comparison purposes. Odds ratio and 95% CI are presented graphically. The minor allele (calculated within each ancestry) was the alternative effect allele. Source data are provided as a Source Data file.

We found 7 significant female-only associations in EUR ancestry. All 7 had significant interactions (mtSNV × sex) across sex groups, demonstrating significantly different effect sizes across females and males (Fig. 2, Top Panel). We also found 3 significant female-only associations in AFR ancestry, 6 male-only associations in AFR, and 3 male-only associations in AMR.

In the female EUR subgroup, a rare noncoding variant, 1555 A > G (MAF = 0.1%), was associated with both carditis (PheCode 420) and congestive heart failure (CHF) (PheCode 428.1). The prevalence of carditis in minor-allele-carriers is 14.7% vs. 2.6% in non-minor-allele-carriers; for CHF, the frequencies are 17.6% vs. 4.5% (Supplementary Data 6A). The mtSNV MT1555A > G is a recognized cause of mitochondrial diseases and nonsyndromic deafness^14–16^. More intriguingly, several case reports have linked this mutation to cardiomyopathy^17–19^. This variant has also recently been reported to be associated with reduced height in UKBB^4^. We could not replicate these associations due to low case incidences in UKBB. However, to further investigate these associations, we used MVP’s well-phenotyped heart failure (HF) phenotypes^20^ for all-cause HF, Heart failure with preserved ejection fraction (HFpEF), and Heart Failure with Reduced Ejection Fraction (HFrEF) subtypes to conduct additional analysis between MT1555A > G and HF (Supplementary Methods). The estimated risks (OR) for HF, HFpEF, and HFrEF are shown in Supplementary Data 6B. We found an association between MT1555A > G and HFrEF, with an OR (95%) of 5.75 (1.93–17.1) and a *p*-value of 1.7e-3, but not with HFpEF. The concordance between HF phenotypes and PheCodes 420 and 428.1 are shown in Supplementary Data 6C.

Also in the female EUR subgroup, mtSNV MT3316G > A (MAF = 0.31%), a missense mutation (A4T) in gene *MT-ND1*, was associated with “other disorders of metabolism” (PheCode 277) (OR, 95% CI, 4.27 (2.18–8.39); *p*-value = 2.46e-5). This variant has been associated with higher fasting blood glucose levels^21^ and diabetes^22,23^. Mutation MT9804G > A in gene *MT-CO3*, a candidate Leber Hereditary Optic Neuropathy (LHON) mutation^24^, was associated with “other abnormal glucose” (PheCode 250.42), and in an expanded PheWAS was also associated with “fracture of unspecified bones” (Supplementary Fig. 9). Unfortunately, we could not replicate these associations due to a low case incidence or lack of genotype data in UKBB. Additional associations in this subgroup included MT2218C > T (*MT-RNR2*) with “encounter for long-term (current) use of aspirin” (PheCode 457.3), MT10044A > G with both “disorders of magnesium metabolism” (PheCode 275.3) and “malaise and fatigue” (PheCode 798, identified through extended PheWAS Supplementary Fig. 9), and MT12937A > G with “noninfectious disorders of lymphatic channels” (PheCode 450). MT10044A > G in *MT-TG* (mt-tRNAGly) has been shown to impair tRNA processing and mitochondrial translation^25^. Because fatigue/malaise are common in mitochondrial diseases^26^ and mitochondria are central to cellular Mg²⁺ homeostasis^27^, their associations with fatigue and magnesium disorders are biologically plausible but remain unconfirmed. MT12937A > G (*MT-ND5*, missense M201V) is generally classified as a benign or haplogroup-linked polymorphism. Although electrolyte abnormalities (including hypomagnesemia) and mitochondrial influences on lymphatic endothelial biology are described at the pathway level^28,29^, neither MT12937A > G nor MT9804G > A has been directly linked to these phenotypes in prior variant-level studies; thus, these associations should be considered hypothesis-generating pending replication with ancestry and medication adjustments.

In AFR females (Fig. 2, Bottom Panel), MT1694T > C (MAF = 0.42%) was associated with both “disorders of the parathyroid gland” (PheCode 252) and “hypovolemia” (PheCode 276.5). MT11969G > A (MAF = 6.2%) was associated with “other venous embolism and thrombosis” (PheCode 452).

In AFR males (Fig. 2, Bottom Panel), we identified six male-specific associations. The start-codon variant MT12338T > C in *MT-ND5* was associated with increased risk of glucocorticoid deficiency (PheCode 255.21) in both the male-only and overall AFR analyses, although the adjusted *p*-value in the overall analysis was marginal after multiple testing. This variant has been described as “possibly pathogenic” and reported in association with Leber hereditary optic neuropathy (LHON)^30^ and aminoglycoside-induced and nonsyndromic deafness^31^. MT10398 is a well-characterized missense mutation (T114A) in the *MT-ND3* gene. MT10398A (minor allele in AFR ancestry) in AFR males was associated with other forms of “chronic heart disease” (PheCode 414) with OR (95% CI) 1.23 (1.12-1.35), *p*-value = 1.45e-05. Allele-frequency differences across ancestries matched prior reports, with 10398 A less common in AFR than EUR^32^ (Table 2). The variant MT10398G (minor allele in EUR ancestry) has been reported to be a protective factor for the onset of Parkinson's disease in EUR ancestry^33^. In contrast, the MT10398A allele was suggested to increase T2D susceptibility in the north Indian population^34^. Four additional AFR male associations had effect sizes similar to those observed in the overall AFR analysis but showed marginal adjusted *p*-values; among these, “adrenal hypofunction” (PheCode 255.2), a phenotype closely related to glucocorticoid deficiency, was associated with MT10345T > C in *MT-ND3*, and cachexia (PheCode 260.1) was associated with MT827A > G (MAF 0.32%).

In AMR males, MT12937 (MAF = 0.14%) was associated with increased risk of cardiomegaly (Phecode 416). MT11172 (MAF = 0.13%) was associated with an increased risk of “disorders of carbohydrate transport and metabolism” (PheCode 271) and its child trait “intestinal disaccharidase deficiencies and disaccharide malabsorption” (PheCode 271.3). Exploratory extended PheWAS suggested additional infectious, hepatic, dermatologic, musculoskeletal, and renal/urogenital phenotypes for both variants, which we regard as hypothesis-generating (Supplementary Fig. 11).

#### MtSNV associations among participants with diabetes

**Fig. 3:**
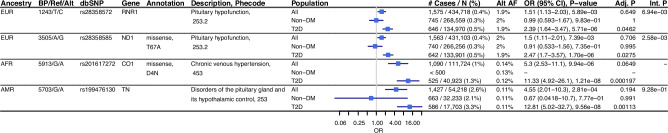
Significant mtSNV associations in T2D across MVP ancestry groups. Participants without diabetes were included for comparison. Firth logistic regression and two-sided *p*-values based on Wald Z-score were reported. Note, for mtSNVs significantly associated with a phenotype in only one subgroup, but not in the overall group for that ancestry, we analyzed and tested for statistical interactions using logistic regression. Significant associations among subgroups that were also significant in the overall group are not shown. EUR European ancestry, AFR African American ancestry, BP base pair of mtSNVs, REF reference allele, ALT alternative allele, Alt AF alternative allele frequency, CI confidence interval, T2D Type 2 diabetes defined by the presence of T2D phecode, Non-DM participants without any diabetes-related phecode; Adj. *P* *p*-value adjusted by Benjamini–Hochberg procedure, Int. *P* *p*-values for testing mtSNV × DM interaction effects. Interaction analyses (mtSNV × DM) were only conducted if the number of cases and controls was > 500 and mtSNVs’ allele frequencies were > 0.1% (refer to Methods for details), and the corresponding *p*-values were marked as “-”. Odds ratio and 95% CI are presented graphically. The minor allele (calculated within each ancestry) was the alternative effect allele. Source data are provided as a Source Data file.

For participants with T2D, we identified 2 associations in EUR, 1 in AFR, and 1 in AMR ancestry (Fig. 3).

In the EUR ancestry group, the variants MT1243T > C and MT3505A > G were associated with an increased risk of pituitary hypofunction (PheCode 253.2) among patients with diabetes, with ORs (95% CI) of 2.39 (1.64–3.47) and 2.47 (1.70–3.57), respectively. Both variants exhibited significant interaction effects, i.e., T2D × mtSNV, indicating that their effects differed significantly between T2D and participants without diabetes. MT3505, located in the *MT-ND1* gene, is a missense mutation resulting in the T67A amino acid change, and has been previously associated with elevated cholesterol levels in a Swedish cohort^35^.

In AFR ancestry, MT5913G > A (MAF = 0.12%) in gene *MT-CO1* was associated with increased risk of “chronic venous hypertension” (PheCode 453).

In AMR ancestry, the rare variant MT5703G > A (MAF 0.11%) was associated with “disorder of pituitary gland and its hypothalamic control” (PheCode 253). This mutation has been reported in multiple ancestry groups, including AFR, Chinese, and EUR. Both MitoTIP and HmtVAR predict MT5703 to be pathogenic, and the Mitochondrial Disease Nuclear and Mitochondrial Variant Curation Expert Panel^36^ classified it pathogenic, given consistent functional evidence of severe deleterious effects across multiple independent studies and repeated apparent de novo occurrences across individuals of different ancestries. MT5703G > A has also been found to be associated with chronic progressive external ophthalmoplegia (CPEO)^37^ and mitochondrial myopathy^38^. The interaction analysis revealed a nonsignificant *p*-value, although ORs estimated in T2D vs. non-diabetics were 12.81 and 0.67, respectively.

### Mitochondrial gene-based rare variant burden analysis

MtSNVs with MAF ≤ 2% were included for gene-based analysis (Supplementary Data 7). This threshold was chosen to maximize aggregation of the rare variants and match the previous study^4^. Out of 44,646 gene × trait tests within ancestries, overall, and in T2D and sex-stratified groups, we identified 12 associations in EUR, 10 in AFR, 2 in AMR, and 2 in EAS ancestry (Table 4 and Supplementary Fig. 12). Among them, 18 trait associations were from the “endocrine/metabolic” chapter. Five trait associations had a significant mtSNV (with the same trait) within that gene. These included “pituitary hypofunction” (PheCode 253.2) among EUR T2D (MT1243T > C/*MT-RNR1* and MT3505A > G/*MT-ND1*), as well as “disorders of the parathyroid gland” (PheCode 252) and “hypovolemia” (PheCode 276.5) among AFR females (MT1694T > C, *MT-RNR2*), and “other abnormal glucose” (PheCode 250.42) among EUR females (MT9804G > A, *MT-CO3*). After re-performing the SKAT-o tests and controlling for significant mtSNVs, none of the 5 gene-based results remained significant (*P* > 0.13), implying that the mtSNVs were the main driver of these gene-based associations (Supplementary Data 8).

**Table 4:** MtGene-based rare variant burden analysis results

^‡^ Trait was defined in the phenotypes section of the manuscript text and included selected PheCodes, biomarkers, and biomarker categories.

^*^ The number of rare mtSNVs included in each testing gene or t-RNA.

*BP* base pairs, *Adj*. *P*-value p-value adjusted by Benjamini–Hochberg procedure; Two-sided gene-level *P* values based on SKAT-o method were reported.

In EUR ancestry, rare variant burden highlighted several biologically coherent signals. A burden in the mitochondrial tRNA gene set was associated with “primary/intrinsic cardiomyopathies” (PheCode 425.1) in females, and the same gene set was associated with “exophthalmos” (PheCode 242.3), an eye phenotype often linked to hyperthyroidism^39–41^, consistent with previous reports of cardiomyopathy-related mtDNA mutations in mitochondrial tRNA genes^42–45^. *MT-ATP6* burden was associated with “disorders involving the immune mechanism” (PheCode 279) in EUR participants with T2D, while *MT-ND6* burden was associated with “polydipsia” (PheCode 276.8) in the T2D, overall, and male-only EUR groups. Among EUR males, *MT-ND3* burden was associated with two vascular traits, “arterial dissection” (PheCode 442.4) and “blood vessel replaced” (PheCode 459.7). *MT-RNR2* burden was also associated with “acute and subacute necrosis of the liver” (PheCode 573.4) in EUR participants with T2D.

In AFR ancestry, several signals converged on adrenal and cardiometabolic function. A burden of 10 rare variants in *MT-ATP6* was associated with “adrenal hypofunction” (PheCode 255.2; overall and males) and its child code “glucocorticoid deficiency” (PheCode 255.21; males only). These adrenal phenotypes were also linked to single variants in *MT-ND3* (MT10345T > C, adrenal hypofunction) and *MT-ND5* (MT12338T > C, glucocorticoid deficiency; Fig. 2), and “hyperaldosteronism” (PheCode 255.12) was associated with *MT-ND4*. Beyond the adrenal axis, *MT-ATP8* burden was associated with “liver abscess and sequelae of chronic liver disease” (PheCode 571.8) in AFR participants with T2D, *MT-ND5* with “myocardial infarction” (PheCode 411.2) in AFR females, and *MT-RNR2* with “disorders of fluid, electrolyte, and acid-base balance” (PheCode 276) in AFR females.

In non-EUR, non-AFR ancestries, the patterns were more focused. In AMR participants with T2D, the MT-ND5 rare variant burden was associated with hyperlipidemia/lipoid metabolism. In EAS males, *MT-ND6* burden was associated with systolic blood pressure, and *MT-RNR2* burden with LDL levels.

## Discussion

In this study, we conducted a multi-ancestry, biobank-scale analysis of mtDNA–phenotype associations. Extending prior biobank efforts (e.g., the UKBB^4^ and Japanese Biobank^5^), we provide, to our knowledge, the most comprehensive biobank-scale evaluation of ancestry-specific mtDNA risks for disease. Mitochondrial disorders show wide clinical and genetic heterogeneity, and early work established that mtDNA haplogroup background can modify disease expression^46^; population-level analyses further demonstrate haplogroup-specific recurrence of variant^47,48^ and a relative depletion of multiple pathogenic changes in European mtDNA^49^, underscoring the need to interpret mtDNA within an ancestry-aware framework. Despite this, ancestry-specific risk estimates for disease onset and progression have remained limited. Here, we identify three AFR-specific associations—MT462C > T, MT10345T > C (in *MT-ND3*), and MT12338T > C (in *MT-ND5*, start-codon)—with pituitary and adrenal disorders, and one association in AMR ancestry. To our knowledge, these ancestry-specific mtSNV associations have not been reported at a biobank scale and expand the catalog of mtDNA contributions to endocrine phenotypes.

Of the 33 mtSNV associations identified, 23 (69.7%) pertained to conditions within the “endocrine/metabolic” PheCode chapter. This represents a significant enrichment compared to other chapters we examined (with a Fisher’s exact enrichment *p*-value of 0.003). Mitochondria play a pivotal role as the primary source of intracellular energy, producing ATP. The ATP generated via OXPHOS is crucial not only for anabolic processes like protein synthesis and cell division but also for physiological functions such as hormone biosynthesis and secretion. Impaired intracellular hormone production or extracellular hormone secretion often underpins the endocrine dysfunction seen in genetic mitochondrial diseases^50,51^. Indeed, among the endocrine disorders we report (Supplementary Data 5), most of them encompass conditions associated with thyroid (hypothyroidism), pituitary (pituitary hypofunction and anterior pituitary disorder), and adrenal (adrenal hypofunction and glucocorticoid deficiency) glands^50–52^, all of which are known clinical manifestations of endocrine dysfunction in mitochondrial diseases. Unlike other inherited rare mitochondrial disorders, endocrine dysfunction is often treatable. Our findings may inform future screening practices and therapeutic strategies.

We identify an association between several mtSNVs in haplogroup J and hypothyroidism and validate this association in the UK Biobank cohort. We also engaged in rigorous sensitivity analyses. As an initial step, we refined our phenotype definition to exclude secondary or non-autoimmune thyroid conditions, such as thyroid cancer or thyroid surgery. Our analytical approach included stratification by age tertiles. Notably, the incidence of hypothyroidism escalated with advancing age groups. Analyses were also differentiated by sex. The prevalence of hypothyroidism in females was approximately twice that of males. Despite these disparities, allele frequencies were uniformly distributed across both sexes. No discernible age-related differential risks were observed for the mtSNVs (refer to Supplementary Methods, Supplementary Data 9), while estimated effect sizes were slightly higher in females than in males (see Supplementary Data 10). Finally, we evaluated the relationship between these 4 mtSNVs and TSH (Supplementary Data 11A and 11B) among participants without hypothyroidism (PheCode 244) or other related PheCodes (Supplementary Data 5) in UKBB. TSH was extracted from primary care data in UKBB (Supplementary Data 7A), and QC procedures were detailed in the Supplementary Materials^53^. Of the 4 mtSNVs evaluated, MT14798T > C was positively associated with TSH with a significant *p*-value (*P* < 0.05/4). This result further implies the link between mtDNA and thyroid diseases. MT228G > A, a control-region (D-loop) variant, has been associated with cataracts in Latino population^54^. MT10398A > G in *MT-ND3* is a well-studied missense change with broad phenotypic associations and demonstrable effects on complex-I bioenergetics, reactive oxygen species (ROS) generation, and apoptosis in cybrid models, with molecular consequences on *MT-ND3* characterized previously^55,56^. Mechanistically, thyroid hormonogenesis depends on mitochondrial ATP supply and a tightly regulated H₂O₂ milieu for thyroperoxidase-catalyzed iodination; modest shifts in OXPHOS efficiency or redox balance are therefore expected to depress hormone output and raise TSH. Variants marking haplogroup J at complex III (*MT-CYB* MT14798T > C) and at complex I (*MT-ND3* MT10398A > G) can alter bioenergetic coupling and ROS set points, while the D-loop change MT228G > A may tune mtDNA transcription/replication and copy number; together our findings may provide a coherent route from mtDNA variation to elevated TSH and hypothyroidism risk.

Growing evidence links mtDNA variation to cardiac disease^57^. Cardiac mitochondrial biology shows sex dimorphism: mtDNA copy number and functional indices in the heart tend to be lower in females, whereas expression of nuclear genes encoding mitochondrial proteins is higher in males^7^. In MVP, we observed a female-specific association of MT1555A > G in *MT-RNR1* with carditis, congestive heart failure, all-cause heart failure, and HFrEF. Recognized as a pathogenic mtSNV, MT1555A > G is classically associated with aminoglycoside-induced deafness^14–16^; our data provide a large-scale confirmation of the cardiac associations suggested by a prior multi-generational case of maternally inherited cardiomyopathy^17^. Although mechanisms remain to be defined, cybrid experiments show that the MT1555A > G ribosomal change can compromise mitochondrial protein translation, especially with aminoglycoside exposure, and that the degree of decreased protein synthesis and oxygen consumption is modified by nuclear genetic background^58,59^. Analogous nuclear-mitochondrial interactions in cardiomyocytes could plausibly drive energetic deficiency and susceptibility to heart failure phenotypes, potentially contributing to the female-specific signal observed here. Future MVP analyses incorporating medication exposure and nuclear-modifier loci should help resolve these mechanisms.

Additional sex-stratified associations were observed but should be viewed as hypothesis-generating. In EUR females, MT10044A > G in *MT-TG* (tRNA-Gly) was associated with disorders of magnesium metabolism, and MT12937A > G with noninfectious disorders of lymphatic channels; replication in UKBB was limited by low case counts or unavailable genotypes. Notably, MT10044A > G shows impaired mt-tRNA processing in vitro^25^, and electrolyte disturbances, including hypomagnesemia, are reported in mitochondrial disease, with Mg²⁺ essential for mt-tRNA folding and efficient mitochondrial translation^28,51,60^. In AFR males, four additional associations had effect sizes similar to those in the overall AFR analysis but marginal adjusted *p*-values; among these, adrenal hypofunction (closely related to glucocorticoid deficiency) tagged MT10345T > C in *MT-ND3*, and cachexia was also implicated. Although these specific variants have not been linked previously to adrenal or cachectic phenotypes, adrenal insufficiency occurs in mitochondrial disorders^61–63^, and complex-I dysfunction is implicated in skeletal-muscle wasting and cachexia^64^. These observations warrant replication and targeted functional work, including mito-nuclear interaction analyses.

Across 44,646 gene-by-trait tests of mtSNVs with MAF ≤ 2%, we identified 26 associations spanning ancestries, many involving endocrine/metabolic traits. In EUR, two signals (including pituitary hypofunction in T2D) were driven by individual SNVs rather than aggregate gene burden. We also observed previously unreported associations—such as mt-tRNA rare-variant burden with primary cardiomyopathy, consistent with known mtDNA tRNA mutations, along with *MT-RNR2* associations across several traits, signals related to adrenal hypofunction in AFR, liver-related traits among participants with T2D, and *MT-ND5* rare variants associated with hyperlipidemia in AMR individuals with T2D.

Our study has several limitations. Although we implemented strong quality control procedures, including validation using in-house whole-genome sequencing, trios, etc., our genotyping chip contains mostly rare variants, and replicating rare variants is challenging. Secondly, while the diverse ancestry of MVP was an overall strength of our analysis, we were unable to replicate our findings in AFR, AMR, and EAS populations due to the lack of mitochondrial genotyping data in other ethnic groups from other databases. Third, we have relied on definitions of cardiometabolic conditions from PheCodes, which can be error-prone. However, the large size of the MVP cohort may relieve the potential impact of this limitation. Fourth, the enrichment of our genotyping with rare variants prevented high-quality haplotyping and imputation. Since mtDNA is inherited as a single, non-recombining unit, there is effectively no recombination between positions, and variants are inherited in complete haplotypes. Variants along the molecule tend to be perfectly correlated within a haplogroup unless mutation or heteroplasmy has altered one of them. Due to the high mutation rate in mtDNA, repeated independent mutations at the same mtDNA site are fairly common, further reducing the utility of linkage methods in mtDNA. In this analysis, we prioritized the investigation of individual SNPs rather than broader haplogroup-level associations. Consequently, our analysis is limited to the variants present in our genotyping array. Fifth, MVP is a US veteran cohort with predominantly male participants. However, the very large MVP cohort still resulted in many unique phenotype cases occurring in women and allowed the identification of several female-only associations. Future confirmatory studies in more general population cohorts with larger numbers of female participants are needed. Sixth, many of the significant associations we observed were only observed in patients with European ancestry, largely due to improved power with larger sample sizes and higher allele frequencies of these variants in the European population. Finally, we did not assess the impact of nuclear mitochondrial variants on cardiometabolic conditions.

In conclusion, we have developed a workflow for QC and analysis of mtDNA genotypes that yields high-quality variants, primarily rare variants, to facilitate future mtDNA analyses. We have comprehensively investigated mtDNA variations and their associations with cardiometabolic conditions and traits. Our findings expand current understanding of relationships between mtDNA variation and health conditions, including hypothyroidism, heart failure, anterior pituitary disorders, and adrenal hypofunction. Future studies will (1) validate these associations in females and in AFR, AMR, and EAS populations; (2) examine interactions between nuclear and mitochondrial genomes; and (3) assess the impact of heteroplasmy using WGS data.

## Methods

The MVP received ethical and study protocol approval from the VA Central Institutional Review Board (cIRB) in accordance with the principles outlined in the Declaration of Helsinki. All relevant ethical regulations for work with human subjects were followed during the study, and written informed consent was obtained from all participants. All research procedures complied with relevant ethical regulations and were approved by the site-specific IRBs of the Palo Alto VA Health Care System, the VA Tennessee Valley Healthcare System, and the Phoenix VA Health Care System.

### Million Veteran Program (MVP), a multi-ancestry cohort

The MVP is a large cohort of U.S. Veterans recruited from 63 participating Department of Veterans Affairs (VA) medical facilities. Recruitment started in 2011, and all Veterans were eligible for participation^65^. We analyzed clinical data for participants who enrolled between 2011 and 2021. Blood samples were collected and banked at the VA Central Biorepository in Boston, where DNA was extracted and shipped to two external centers for genotyping^66^. The electronic health record (EHR) of each veteran was integrated into the MVP biorepository and included inpatient International Classification of Diseases (ICD-9-CM and ICD-10-CM) diagnosis codes, Current Procedural Terminology (CPT) procedure codes, clinical laboratory measurements, and reports of diagnostic imaging modalities. Researchers were provided data that were de-identified except for dates.

MVP is a multi-ancestry cohort. Genetically inferred ancestry (GIA)^67^ was adopted in our analysis. GIA was clustered based on a random forest approach using the 1000 Genomes Project (1KGP) and the Human Genome Diversity Project (HGDP) as reference panels. Four ancestry categories were considered: EUR, AFR, AMR, and EAS. Due to small sample sizes, our analyses did not include Central/South Asian and Middle Eastern ancestries.

### Genotyping and quality control

The MVP cohort was typed using the Axiom MVP 1.0 array. The MVP 1.0 array content is based on the Applied Biosystems Axiom® Biobank core variants. In addition, it includes markers designed to (a) assist imputation for non-European samples; (b) improve HLA imputation; (c) target previously known common and rare disease associations^66^. Only a small fraction of the mitochondrial variants present on the chip reach appreciable frequency in the population. Most mitochondrial variants were selected for inclusion based on literature reporting variants of strong effect in rare mitochondrial diseases. There is, therefore, a significant bias towards low allele frequency variants^66^. Over long evolutionary time scales, copies of the mitochondrial DNA have been transferred into the nuclear genome (noted as nuMTs). Variant sites in nuMTs can be mistaken for very low-level heteroplasmy in mtDNA, but due to the high number of mtDNA copies per nuclear copy (>100) in blood samples^68^, nuMTS do not affect the assessment of fixed mtDNA variants. Basic and advanced marker QC for autosomal and hemizygous markers (chromosome Y and mtDNA) was performed as described in Hunter-Zinck et al.^66^ with one notable difference. For MVP release 4, the data set presented here, 68 mitochondrial single nucleotide polymorphisms (mtSNVs) were used, with marker-specific priors defined by Thermo Fisher. Visual inspection of the cluster plots confirms a significant improvement in the definition of genotype clusters for these mtSNV markers. We verified the validity of mtDNA calls using two additional procedures specific to mtSNVs: concordance between intentional duplicates (30,347 samples typed more than once) and comparison of array results to whole genome sequencing (WGS)-derived genotypes (1,835 samples). Finally, we compared 248 mtSNVs’ allele frequencies calculated in each MVP ancestry group to those reported in Genome Aggregation Database (gnomAD) v4.1^69^. We did not conduct imputation because our genotype platform was enriched with rare variants.

### MtDNA variant annotation

248 mtSNVs genotyped in the MVP were annotated using a programmatic API query of the HmtVar resource^70^. These included mitochondrial locus, variant type, amino acid change (for coding variants only), disease annotations in ClinVar^71^ and MITOMAP databases, and several pathogenicity predictions and conservation scores. We considered pathogenic variants with the indication of association with diseases in both ClinVar^71^ and MITOMAP databases. We further annotated variants with confirmed MITOMAP pathogenic status based on the list provided as of Oct 1, 2023 (https://www.mitomap.org/foswiki/bin/view/MITOMAP/ConfirmedMutations/).

MT####Ref>Alt denotes rCRS coordinate throughout the manuscript.

### Cardiometabolic phenotypes

Cardiometabolic phenotypes included all PheCodes in the endocrine/metabolic and circulatory systems, and select liver- and pancreas-related conditions in the digestive chapter^72^. Each PheCode represents ICD codes grouped into clinically relevant phenotypes for clinical studies and structured hierarchically (Fig. 1). Participants with the PheCode present were defined as cases, whereas those with no instance of a PheCode-mapped ICD-9 or ICD-10 code were defined as controls. Based on previous simulation studies, PheCodes comprising <200 cases were more likely to produce spurious results^73^. Thus, we only included PheCodes with at least 500 cases among AFR, AMR, EAS, and EUR groups for analysis. We analyzed 316 PheCodes: 137 in the endocrine/metabolic chapter, 161 in the circulatory system chapter, and 18 in the digestive chapter related to the above conditions. T2D for subgroup analysis was defined as PheCode 250.2 or 250.2x in the absence of PheCode 250.1 or 250.1x (T1D); non-DM participants were defined as those without T2D or T1D. Heart failure (HF), heart failure with preserved ejection fraction (HFpEF), and heart failure with reduced ejection fraction (HFrEF) analyses were discussed in the Supplementary Methods^20^.

We also analyzed cardiometabolic-related quantitative traits, including body mass index (BMI), systolic blood pressure (SBP), diastolic blood pressure (DBP), hemoglobin A1c (HbA1c), high-density lipoprotein (HDL) cholesterol, low-density lipoprotein (LDL) cholesterol, and triglyceride levels extracted from VA EHR data close to participants’ time of recruitment. Obesity, as a binary trait, was defined as body mass index (BMI) > 30 kg/m^2^. To adjust for the effect of statin treatment on the quantitative lipid level trait, we added 18.41 mg/dL (0.208 mmol/L) to patients’ triglyceride levels, 49.88 mg/dL (1.290 mmol/L) to LDL cholesterol, and subtracted 2.32 mg/dL (0.060 mmol/L) from HDL cholesterol^74^ for patients taking statins. We inverse normal transformed each lipid trait. We added 15 mmHg and 10 mmHg to SBP and DBP, respectively, for patients on anti-hypertensive medications^75^. Other continuous outcome variables were not transformed. HbA1c analysis was only performed stratified by diabetes status^76^.

### Statistical analysis

The overall analysis approach is summarized below and in Fig. 1:

- We performed mtSNV and mitochondrial gene-based rare variant burden association analysis in ancestry-stratified, sex-stratified, and diabetes-status-stratified groups. For both single variant and gene-based association analyses, we adjusted for sex (except in the sex-stratified analysis), age, and 15 genome-wide principal components (PCs). We excluded patients with ambiguous genetically-inferred sex or whose reported sex did not match their genetically-inferred sex.
- The significance level was controlled at a false discovery rate (FDR) of 5% for the number of mitochondrial SNVs or genes and trait pairs tested in each population by the Benjamini-Hochberg (BH) procedure^77^. The number of tests performed in each population varied according to allele frequencies (MAF > 0.1%) calculated in the testing population and event rates (binary traits tested must have at least 500 cases and 500 controls); thus, the significance threshold also varied by population.
- We attempted to replicate our mtSNV findings in EUR ancestry in the UK Biobank (UKBB) study. We did not attempt to replicate rare variant burden analysis, as rare variant sets may differ across studies due to the differences in allele frequencies and the design of the genotyping platform. For example, most UKBB genotyped mitochondrial variants are common with MAF > 5%.
- For all reported significant mtSNVs, we conducted extended PheWAS analyses within the corresponding population, including PheCodes from all the remaining chapters (Supplementary Methods).
- To help with the interpretation of each reported PheCode and its clinical relevance, we extracted and reported its co-occurring PheCodes/labs/medications and their cosine similarities extracted by the KESER (Knowledge extraction via sparse embedding regression, https://celehs.connect.hms.harvard.edu/kesernetwork/) network trained in VA EHR and Mass General Brigham (MGB) database^13^ (Supplementary Methods).
- We selected two identified associations (hypothyroidism and congestive heart failure) for detailed *post hoc* investigations (Supplementary Methods).

#### MtSNV association analyses

To avoid the potential impact of imbalance in case and control proportions, mtSNV association analyses were conducted using Firth logistic regression by the Plink2.0 package (--glm function with firth option specified)^78^, and two-sided *P* values based on Wald Z-score were reported. Variants with population-specific MAF > 0.1% and call rate ≥98% were selected for mtSNV analysis. Samples that were missing more than 2% of their mitochondrial genotype calls were excluded. We stratified our analysis within each ancestry group to compare the directionality of associations between EUR and other ancestries. For mtSNVs that were significantly associated with a phenotype in one subgroup but not in the overall group for that ancestry, we analyzed and tested for statistical interactions with the relevant stratifying variable (T2D status or sex) using logistic regression. We required inclusion criteria to be met in both strata (≥ 500 cases, ≥500 controls, MAF ≥0.1%, and variant call rate ≥98%). Power considerations were included in the Supplementary Materials. Cross-ancestry meta-analyses were not conducted due to the enrichment of rare variants in our mtSNVs and potential dramatic differences in allele frequencies across ancestries.

#### Mitochondrial gene-based rare variant association analysis

MtSNVs were selected for gene-based rare variant (MAF ≤ 2%) testing. Genes with at least 2 genotyped rare variants were kept for analysis using the SKAT-o method in the R package, and two-sided gene-level *P* values were presented^79,80^. We considered 14 mtSNV sets/groupings (i.e., 13 genes, and all tRNAs as a single set). For significant gene-based associations where an underlying mtSNV was also significantly associated with the same trait, we performed an additional SKAT-o test for the gene-trait pair while controlling for the genotype of the significant SNP to assess whether the SNP was the main driver of the association at the gene level.

#### Replication in UKBB

We attempted to replicate significant mtSNV associations among those of white British descent in the UKBB^81^, for associations found in EUR ancestry in MVP, including from sex- and diabetes-status-stratified analyses. A 2-step approach was adopted to reproduce PheCodes in UKBB: (1) Using the PheWAS package^72^ and its ICD10-to-phecode map, we generated the PheCode phenotype using ICD10 codes from UKBB hospitalization records, and performed the association tests; (2) If the case proportion in UKBB was less than 1/4 that of MVP and the replication *P*-value was not significant, we searched the UKBB “First Occurrence” fields (Category 1712, Resource 593) for corresponding phenotypes. We utilized genetically inferred sex for sex-stratified analyses; those with ambiguous genetic sex were excluded. For diabetes-status-stratified analyses, T2D patients were defined as those with any self-report of, or diagnosis code for, T2D, but none for T1D. Non-DM patients were those without any self-report or diagnosis code for diabetes of either type.

### Reporting summary

Further information on research design is available in the Nature Portfolio Reporting Summary linked to this article.

## Supplementary information

Supplementary Information

Description of Additional Supplementary Files

Supplementary Data 1-13

Reporting Summary

Transparent Peer Review file

## Source data

Source data

## Data Availability

The summary statistics generated in this study from the Million Veteran Program (MVP) are provided in the Supplementary Data and Source Data files. The summary statistics from MVP are available via the dbGap study accession number phs001672 (Veterans Administration MVP Summary Results from Omics Studies). Individual-level MVP data are protected and not publicly available due to participant privacy restrictions. Source data are provided with this paper.

## Code availability

Code for data cleaning and for reproducing the manuscript tables and figures is available at https://github.com/jinjinzhou/mtDNA/ and archived at Zenodo (10.5281/zenodo.18654157)^82^.
