# Supplementary material for: A large-scale multi-ancestry mitochondrial variant association analysis for cardiometabolic traits
Source: Nat Commun. 2026 Jul 4;17:8319. doi: 10.1038/s41467-026-74939-4 (PMC13469969; doi:10.1038/s41467-026-74939-4)
Supplement: Supplementary file 1 [file 41467_2026_74939_MOESM1_ESM.pdf]

Supplementary Materials

"A large-scale multi-ancestry mitochondrial variant association analysis for cardiometabolic traits"

Table of Contents

Supplementary Figures **2**

Supplementary Figure 1. Cohort generation flowchart. **2**

Supplementary Figure 2. Genotype call rate distributions for 30,347 intentional duplicates across WGS and MT marker array. **3**

Supplementary Figure 3. Non-Reference-Discordance (NRD) distributions shown as a histogram (left) and boxplot (right) for genotype comparisons across 30,347 intentional WGS and MT array marker duplicates. **4**

Supplementary Figure 4. Non-Reference-Discordance (NRD) versus call rate (left) and allele frequency distributions (right) for genotype comparisons across 30,347 intentional WGS and array MT marker duplicates. **5**

Supplementary Figure 5. Whole-genome sequencing heteroplasmy estimates for 1,835 subjects. **6**

Supplementary Figure 6. Non-Reference-Discordance (NRD) by sample and by genotypes between WGS and array MT markers for 1835 pairs. **7**

Supplementary Figure 7. MVP mitochondrial allele frequency distribution (across all ancestries) compared with those in UK Biobank (UKBB), 1000Genome (G1K), and HmtDB. **8**

Supplementary Figure 8. Minor allele frequency (MAF) comparison between MVP and gnomAD databases calculated using homoplasmic variants. **9**

Supplementary Figure 9. Extended PheWAS in the EUR ancestry **10**

Supplementary Figure 10. Extended PheWAS in the AFR ancestry **11**

Supplementary Figure 11. Extended PheWAS in the AMR ancestry **12**

Supplementary Figure 12. MtGene rare variant burden associations in different ancestry groups and subgroups. **13**

Supplementary Methods **14**

Genotyping and quality control **14**

Power considerations **15**

Extended PheWAS **16**

KESER network analysis **17**

Sensitivity analysis for hypothyroidism **18**

MtDNA and thyroid-stimulating hormone (TSH) in UKBB **19**

MT1555 and heart failure in MVP **20**

References **21**

VA Million Veteran Program: Core Acknowledgment for Publications **22**

Supplementary Figures

Supplementary Figure 1. Cohort generation flowchart.

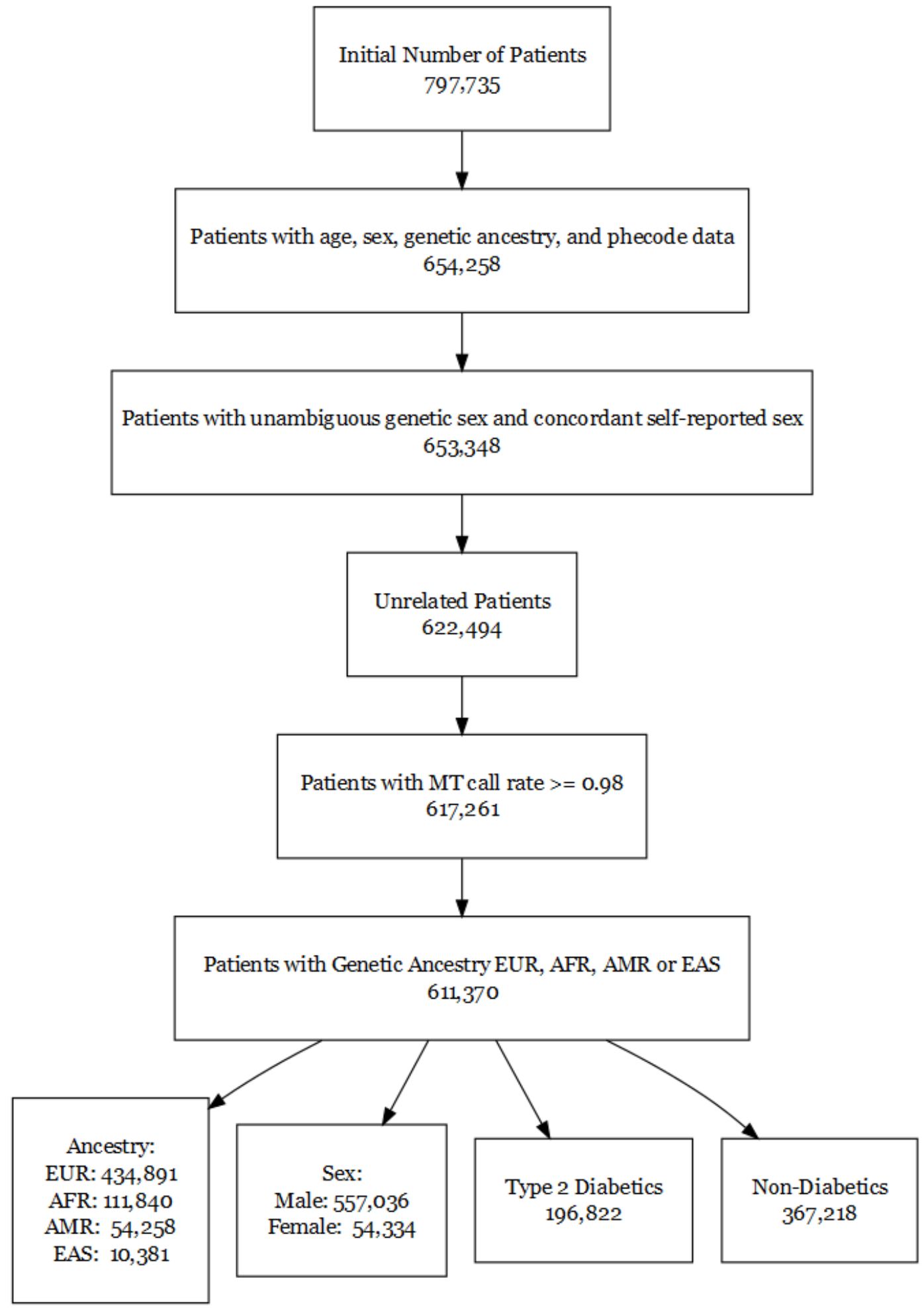

MT: mitochondrial; EUR: European ancestry; AFR: African ancestry; AMR: Admixed American ancestry; EAS: East Asian ancestry. Source data are provided in the Source Data file.

Supplementary Figure 2. Genotype call rate distributions for 30,347 intentional duplicates across WGS and MT marker array.

Call rate distributions were shown using two different x-axis scales: (0.7, 1) (left) and (0.95, 1) (right).

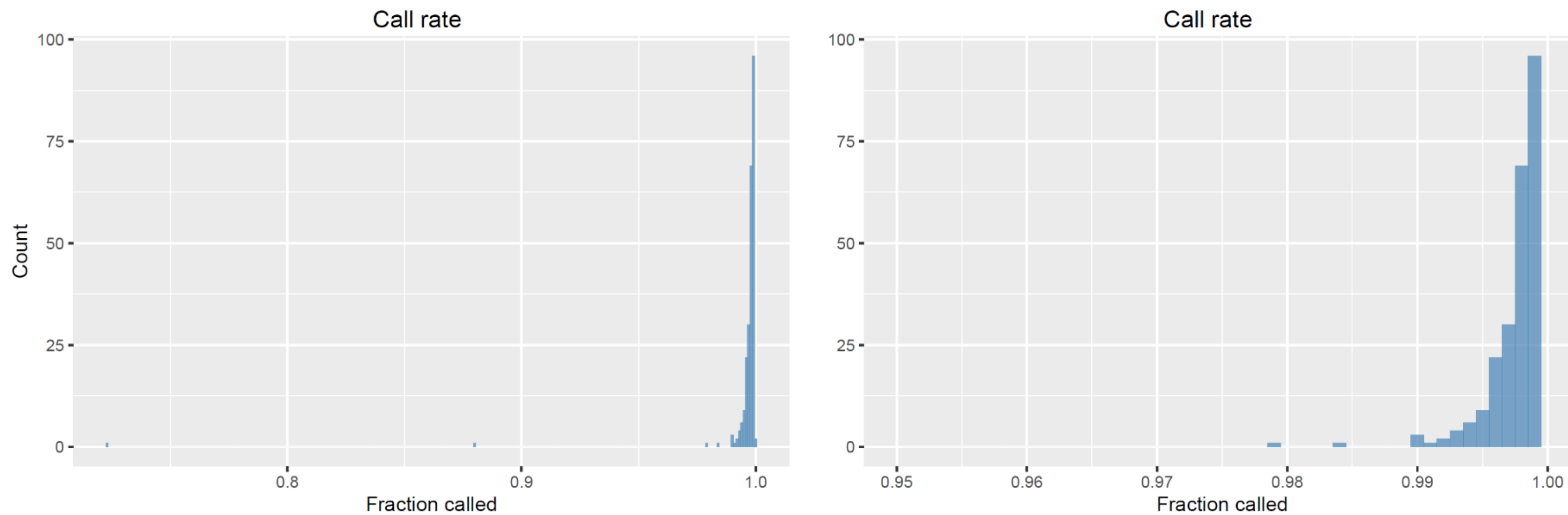

WGS: Whole Genome Sequencing; MT: mitochondrial. Source data are provided in the Source Data file.

Supplementary Figure 3. Non-Reference-Discordance (NRD) distributions shown as a histogram (left) and boxplot (right) for genotype comparisons across 30,347 intentional WGS and MT array marker duplicates.

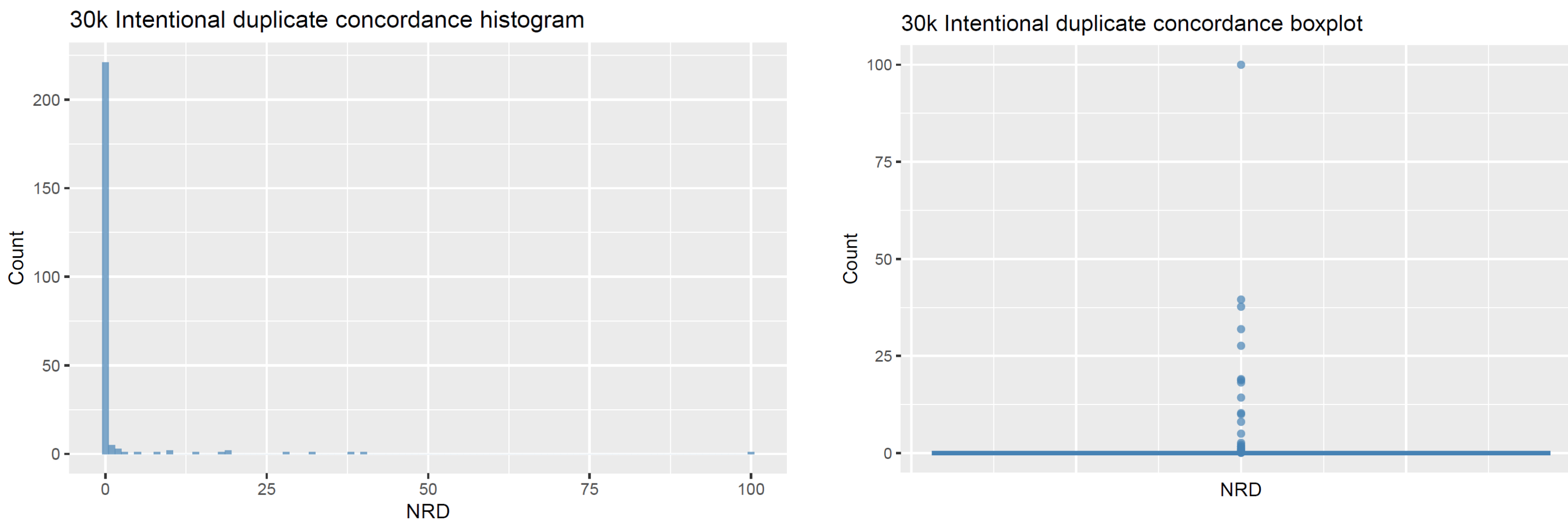

WGS: Whole Genome Sequencing; MT: mitochondria. Source data are provided in the Source Data file. Source data are provided in the Source Data file.

Supplementary Figure 4. Non-Reference-Discordance (NRD) versus call rate (left) and allele frequency distributions (right) for genotype comparisons across 30,347 intentional WGS and array MT marker duplicates.

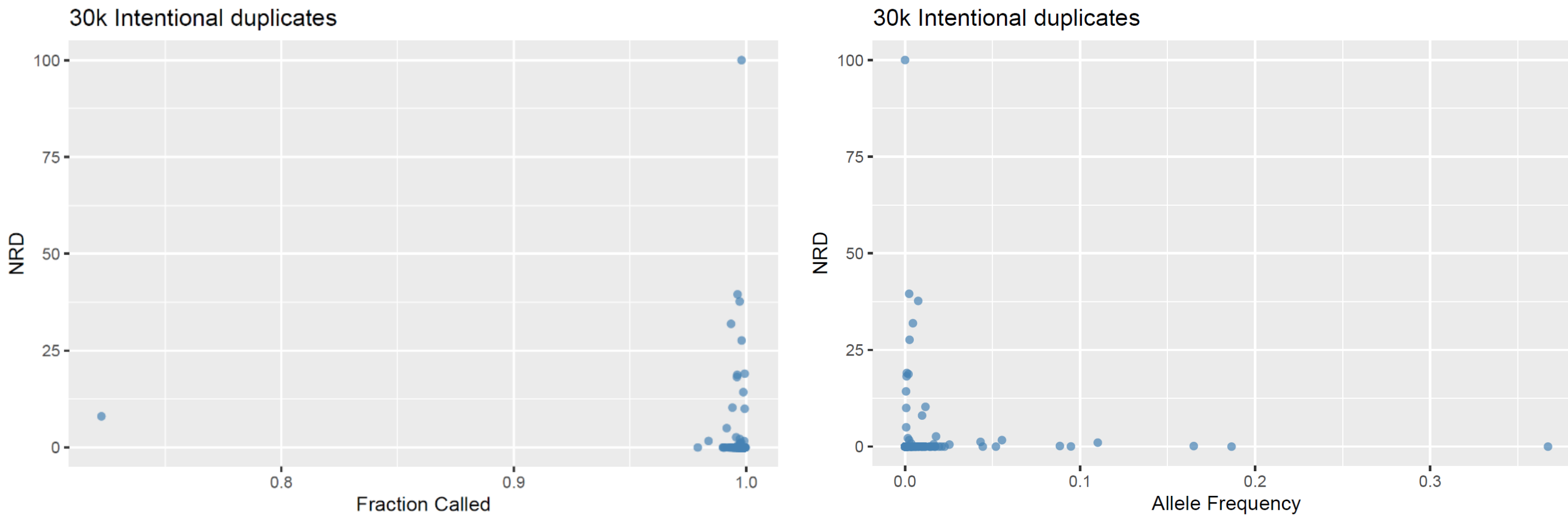

WGS: Whole Genome Sequencing; MT: mitochondria. Source data are provided in the Source Data file.

Supplementary Figure 5. Whole-genome sequencing heteroplasmy estimates for 1,835 subjects.

Note that the same distribution is shown for three x-axis ranges.

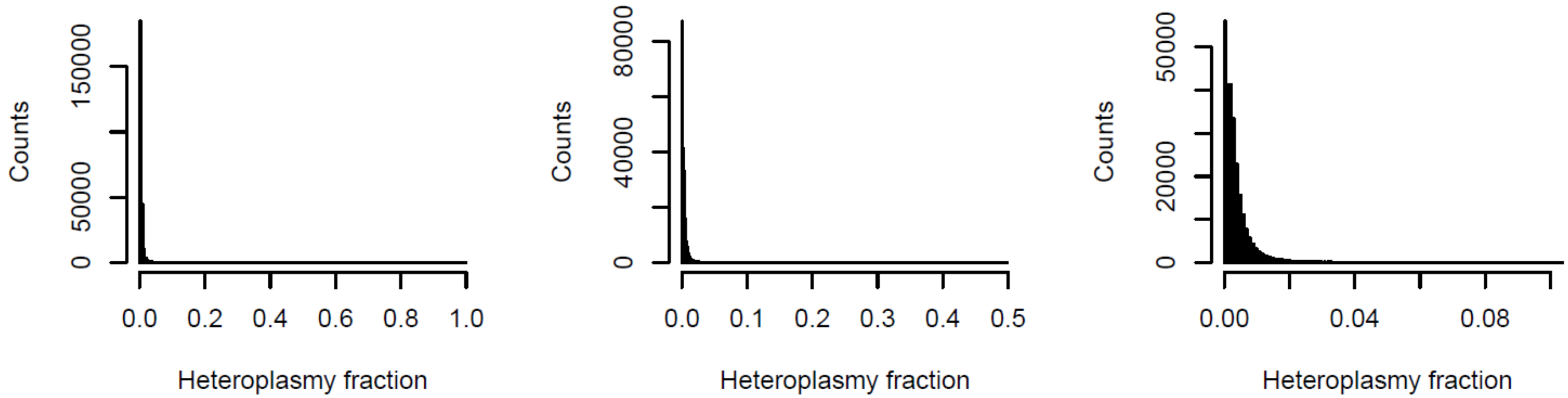

Supplementary Figure 6. Non-Reference-Discordance (NRD) by sample and by genotypes between WGS and array MT markers for 1835 pairs.

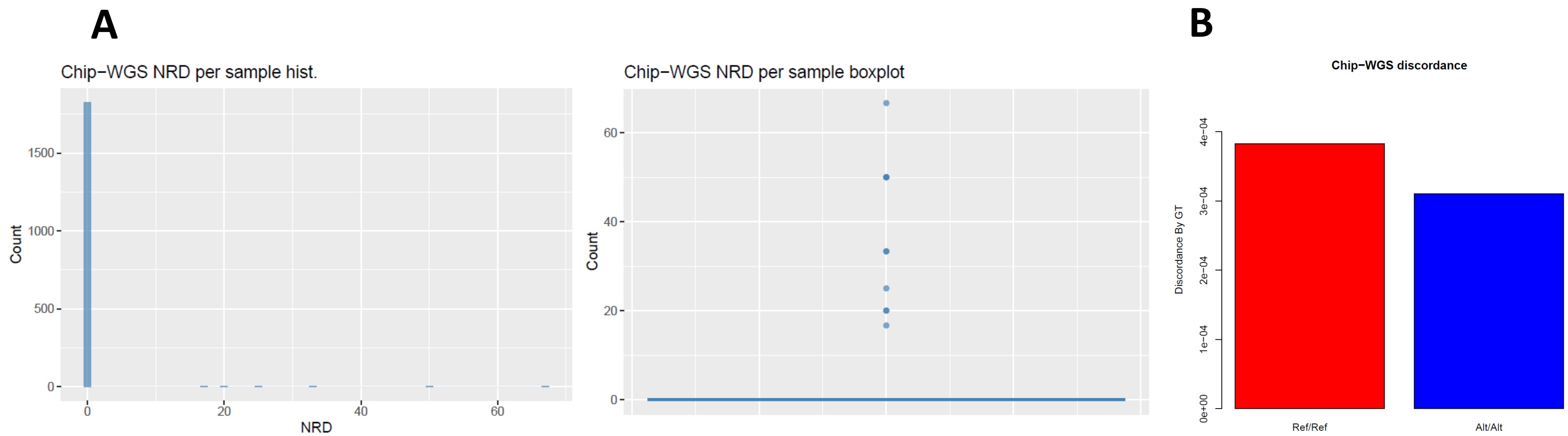

WGS: Whole Genome Sequencing; MT: mitochondria. Source data are provided in the Source Data file.

Supplementary Figure 7. MVP mitochondrial allele frequency distribution (across all ancestries) compared with those in UK Biobank (UKBB), 1000Genome (G1K), and HmtDB.

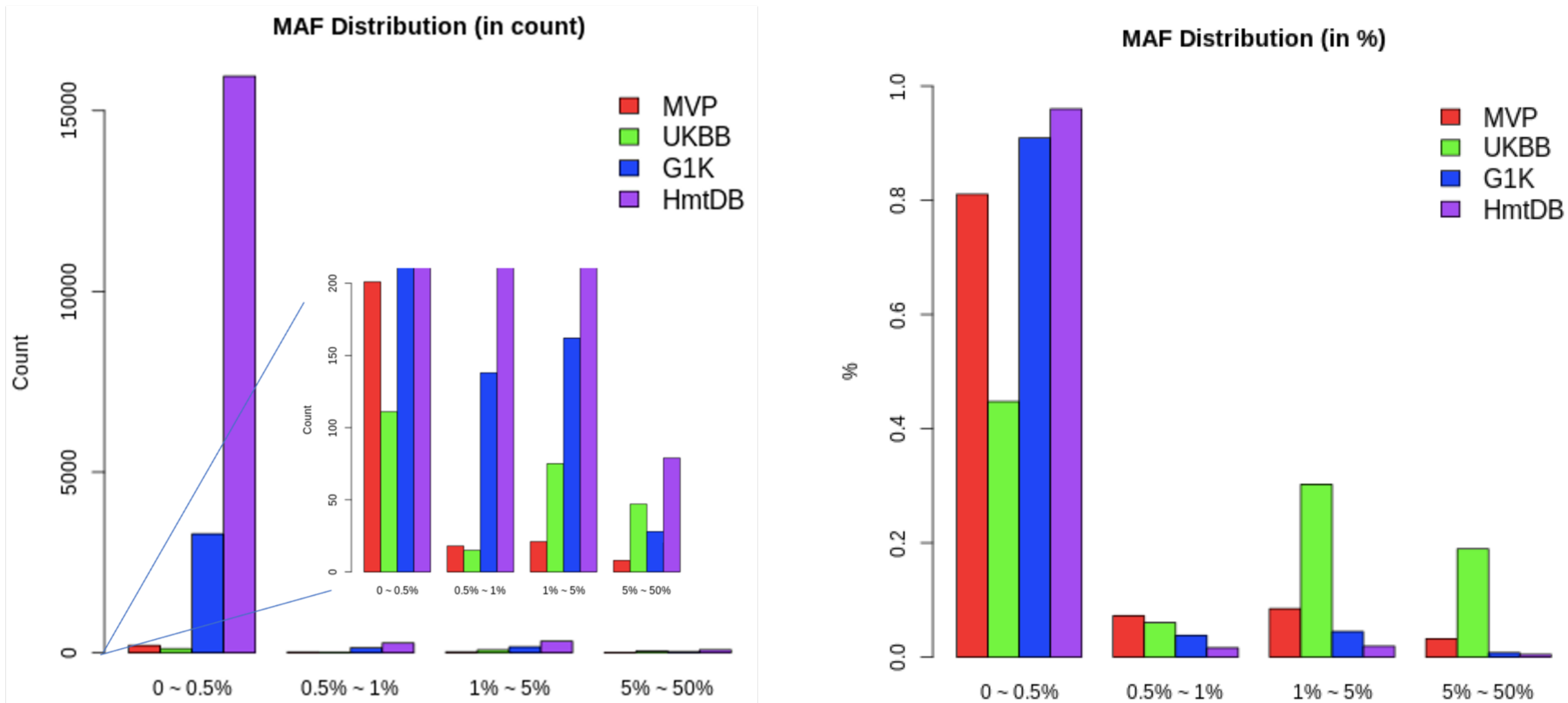

Supplementary Figure 8. Minor allele frequency (MAF) comparison between MVP and gnomAD databases calculated using homoplasmic variants.

The estimated r^2 and slope were annotated in each graph.

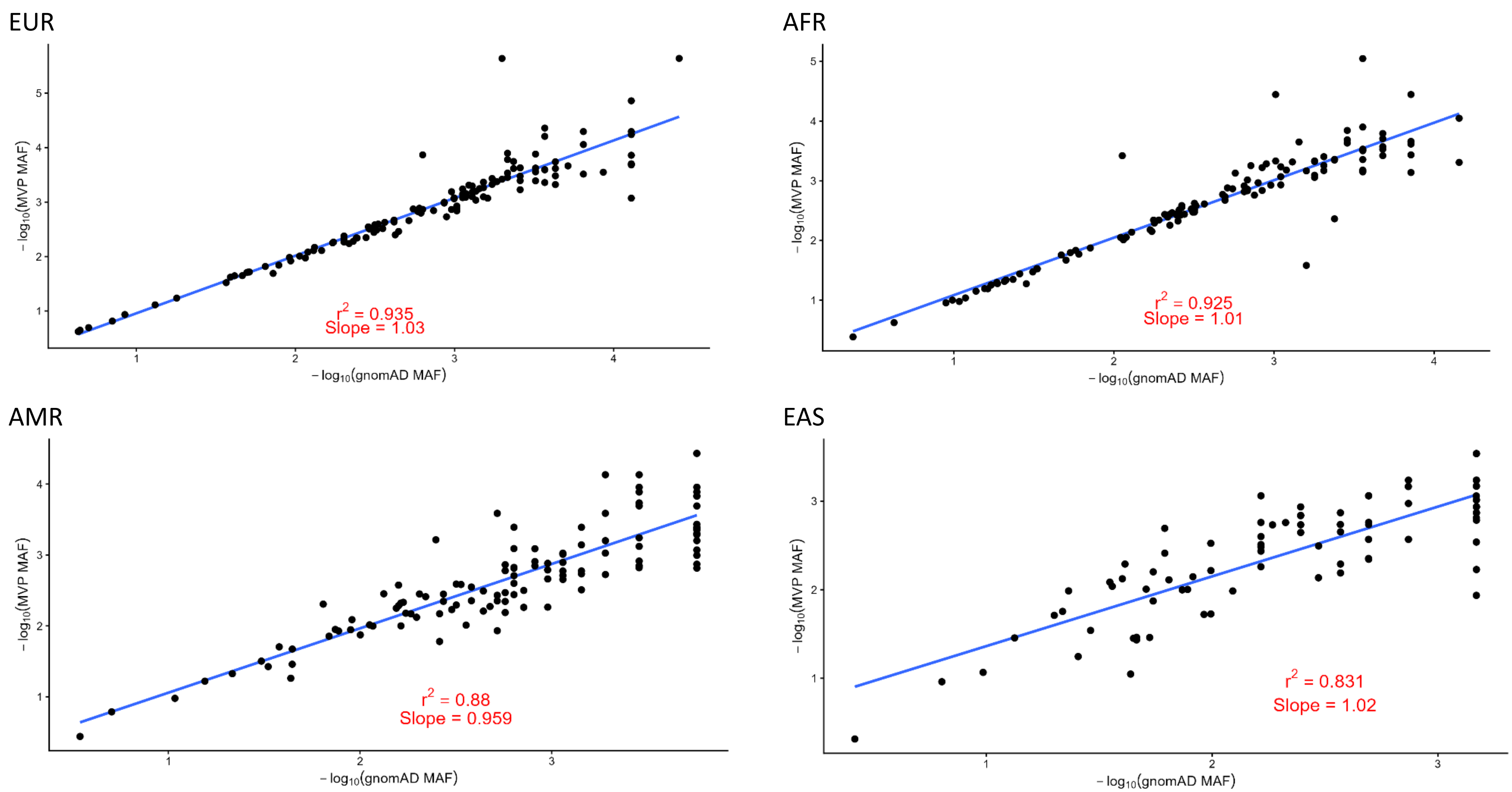

MVP: Million Veteran Program; gnomAD: The Genome Aggregation Database; EUR: European ancestry; AFR: African ancestry; AMR: Admixed American ancestry; EAS: East Asian ancestry. EUR MAF in MVP is compared to Non-Finnish Europeans (NFE) in gnomAD. Source data are provided in the Source Data file.

Supplementary Figure 9. Extended PheWAS in the EUR ancestry

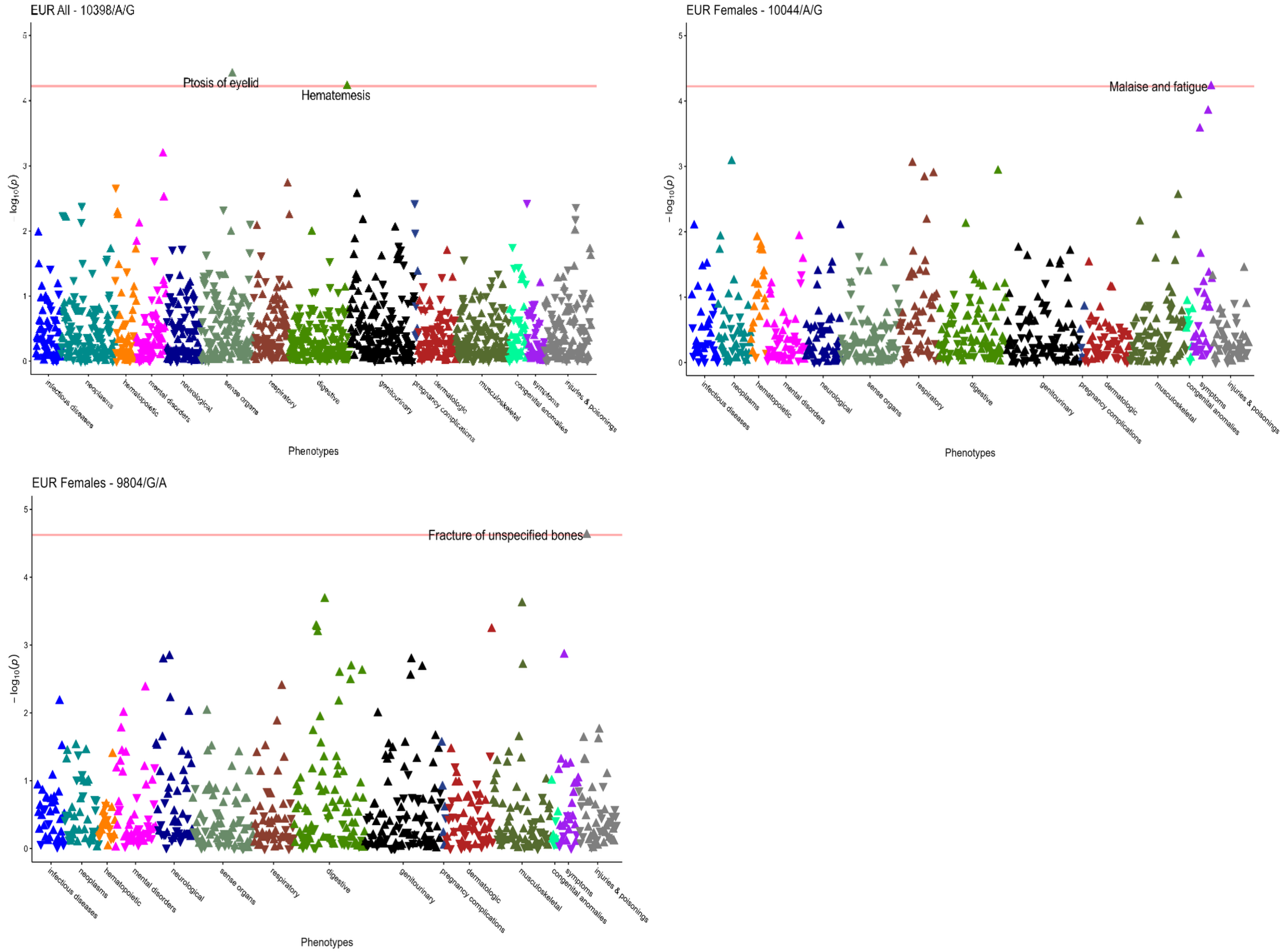

The False Discovery Rate (FDR) P-value threshold for all patients of European (EUR) ancestry for variant 10398/A/G was 6e-5. The FDR threshold for female patients of EUR ancestry for variants 10044/A/G and 9804/G/A were 6e-5 and 2.4e-5, respectively. Source data are provided in the Source Data file.

Supplementary Figure 10. Extended PheWAS in the AFR ancestry

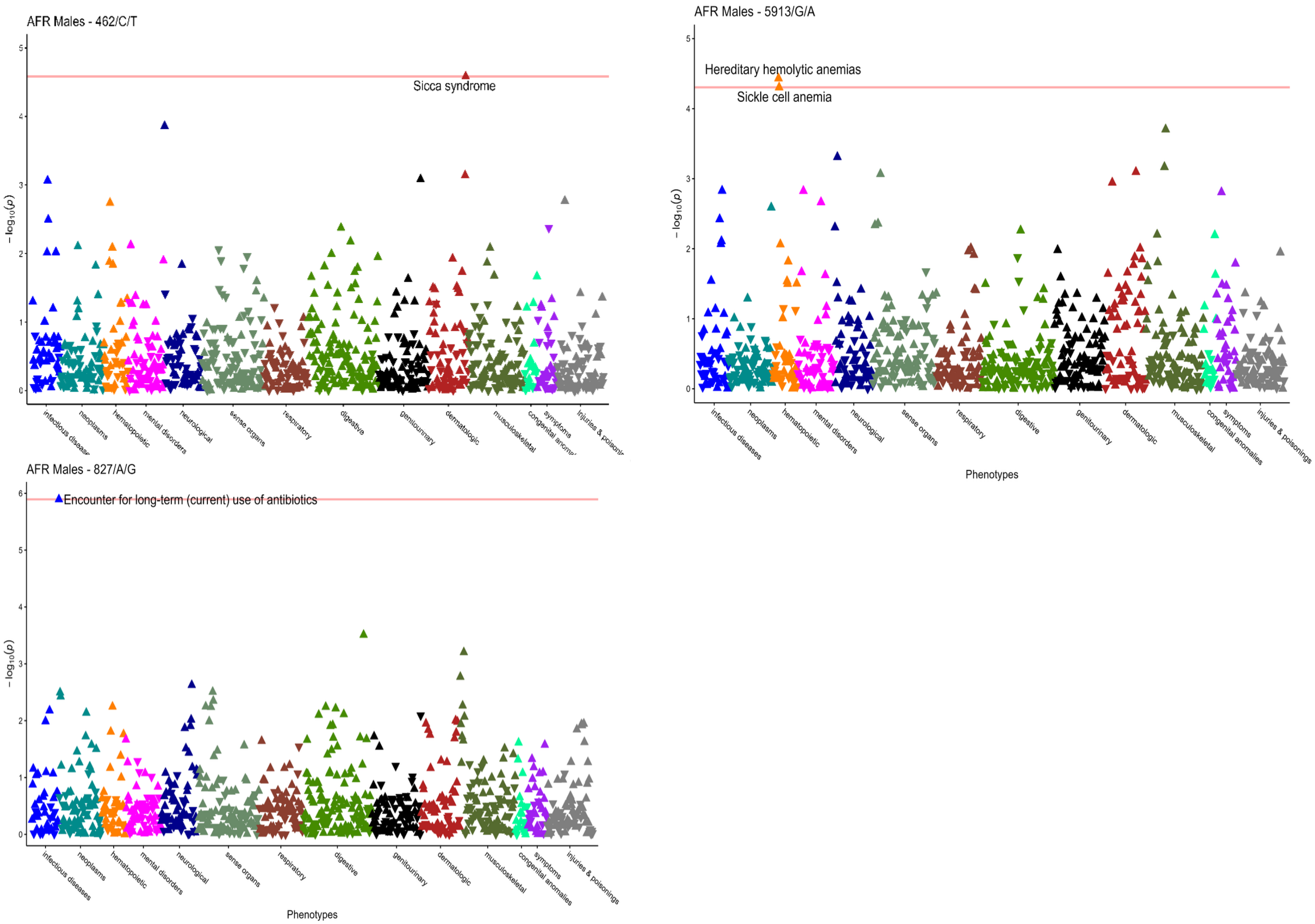

The False Discovery Rate (FDR) P-value threshold for male patients of African (AFR) ancestry for variants 5913/G/A, 462/C/T, and 827/A/G were 5e-5, 2.6e-5, and 1.3e-6, respectively. Source data are provided in the Source Data file.

Supplementary Figure 11. Extended PheWAS in the AMR ancestry

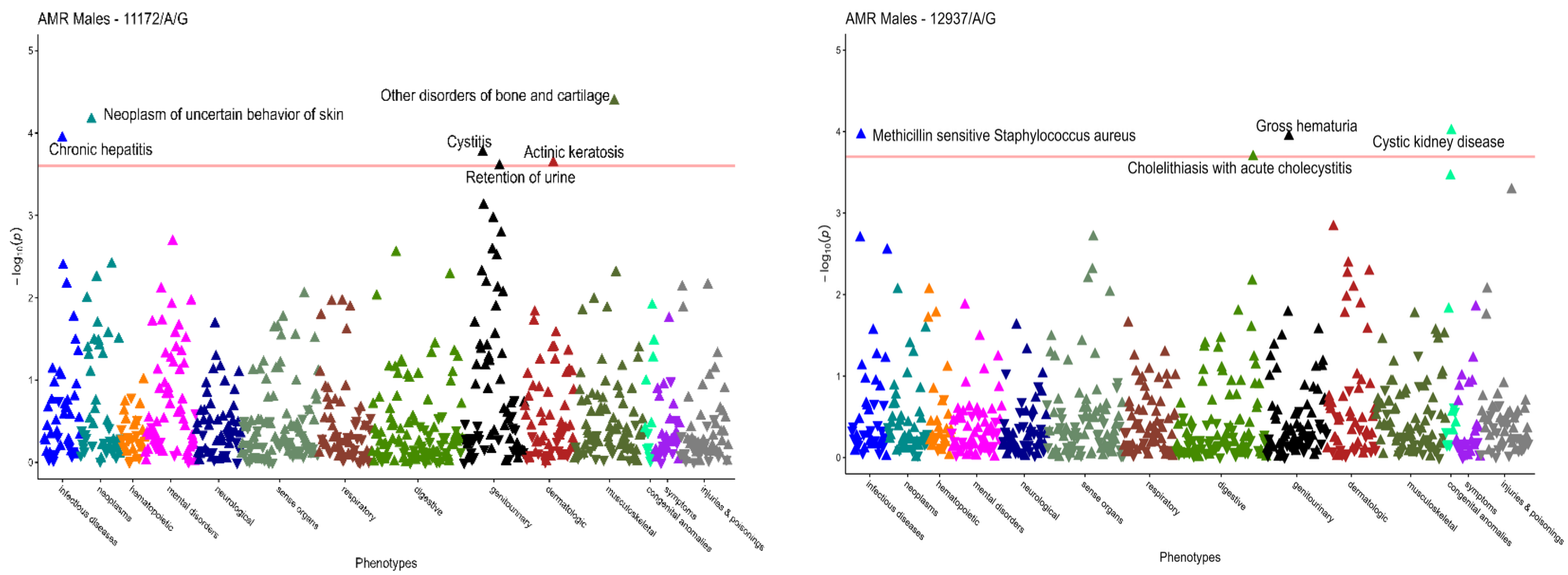

The False Discovery Rate (FDR) threshold for male patients of Admixed American (AMR) ancestry for variants 11172/A/G and 12937/A/G were 2.5e-4 and 2.0e-4, respectively. Source data are provided in the Source Data file.

Supplementary Figure 12. MtGene rare variant burden associations in different ancestry groups and subgroups.

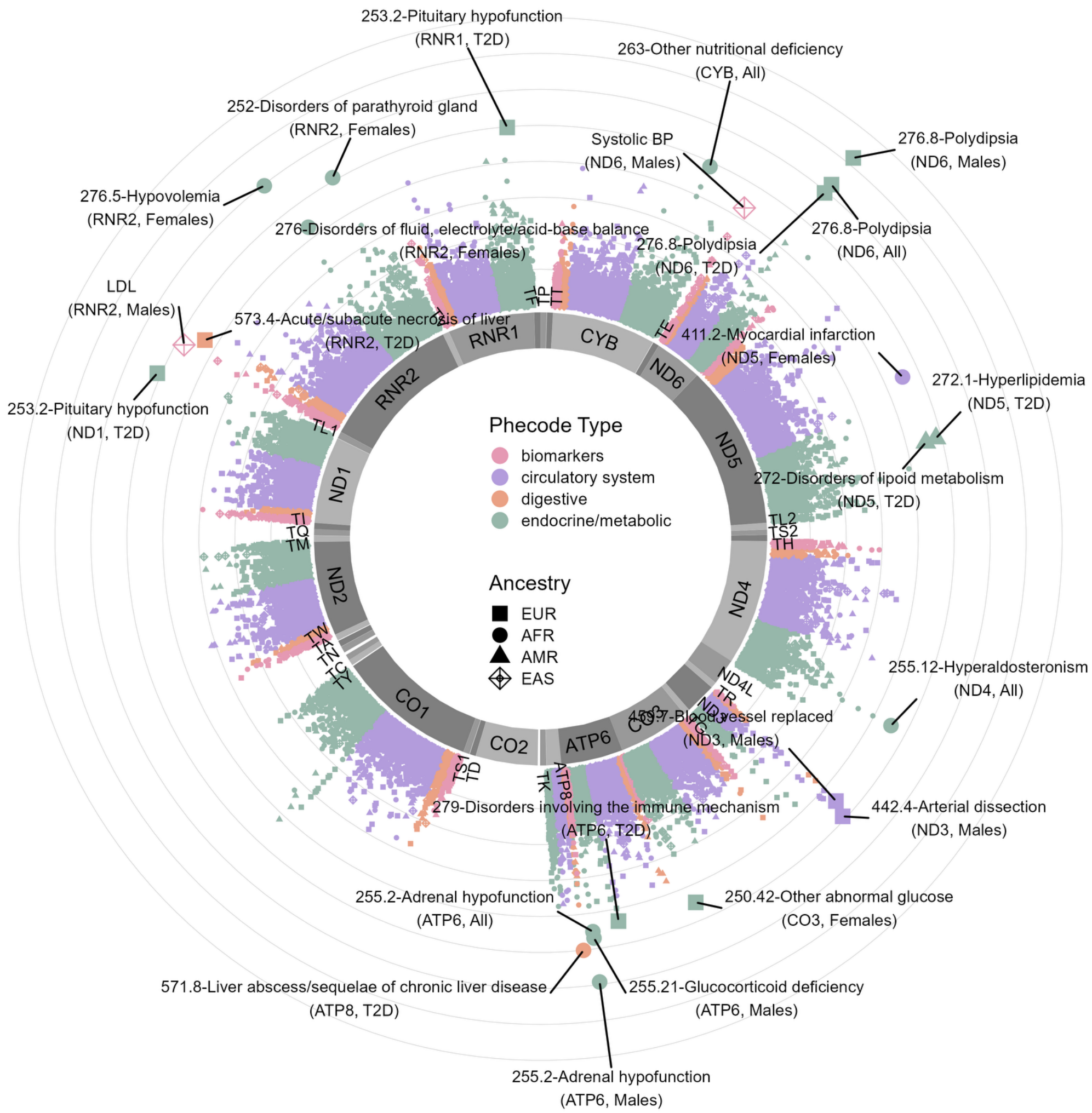

EUR: European ancestry; AFR: African ancestry; AMR: Admixed American ancestry; EAS: East Asian ancestry. Source data are provided in the Source Data file.

Supplementary Methods

Genotyping and quality control

Concordance analysis. For genotype duplicates and genotype-WGS comparisons we define the metric Non-Reference-Discordance (NRD) as:

NRD = (xRR + xRA + xAA) / (xRR + xRA + xAA + mRA + mAA)

where RR: ref/ref; RA: ref/alt; AA: alt/alt; x: mismatch; m: match. The metric omits the matching reference counts (mRR) in the denominator to highlight discordance for rare markers. For both comparisons we used bcftools after conversion of genotypes to diploid calls.

Intentional duplicates. Concordance between intentional duplicates confirms the consistency of the genotyping assay. There were 30,347 intentional duplicates from release 4. Out of 248 MT markers, 89.7% of markers have NRD = 0 (Supplementary Figure 3). NRD is not associated with low call rate, however, high NRD markers have low allele frequency (Supplementary Figure 4). In summary, 98.43% of samples have perfect NRD, and 89.7% of markers have perfect NRD.

Genotype-WGS. WGS variant calling was performed using MtoolBox with rCRS as the reference. We required low heteroplasmy per marker (i.e., heteroplasmy fraction (HF) < 0.01), and we did not compare multi-allelic markers. Only 2% of markers have HF > 0.01. Among markers with AF > 1%, 8/37 are multiallelic and were not included in the comparison. Out of 121 diallelic non-reference markers in either assay, 116 (95.9%) markers have NRD = 0 (perfect concordance). 99.46% (1825/1835) samples have NRD = 0 (perfect concordance). For genotype-WGS comparison (1835 pairs), we observe NRD < 0.03 for all but 3 markers. For all 3 markers AF < 0.003 and total mismatch counts were 1 or 2 per marker. In conclusion, we confirm consistent calling and verify the quality of genotype calling using WGS for 248 markers mitochondrial markers released to MVP.

Power considerations

We performed power calculations by simulation in each ancestry. In the European population, we had 80% power to detect associations for rare alleles (MAF=0.001) when the OR was ~1.7 and the case proportion was 10%. We had 80% power to detect associations for rare traits (1%) when the MAF was 1% and the OR was 1.6. For both rare alleles and cases (0.001 and 1%), an OR of > 3.3 would be required to detect the association. Other ancestries with smaller population sizes had reduced power to detect associations. In these ancestries, the number of SNP-trait pairs tested tended to be lower, and the typical case proportion higher, due to the requirement of >500 cases of the trait in the specific ancestry group. Most associations identified in European ancestry involved common alleles and traits. All of the reported associations had a predicted power (at alpha = 1e-5) of >75% for EUR, and >69% for AFR, based on the MAF, trait frequency, and sample size of the population.

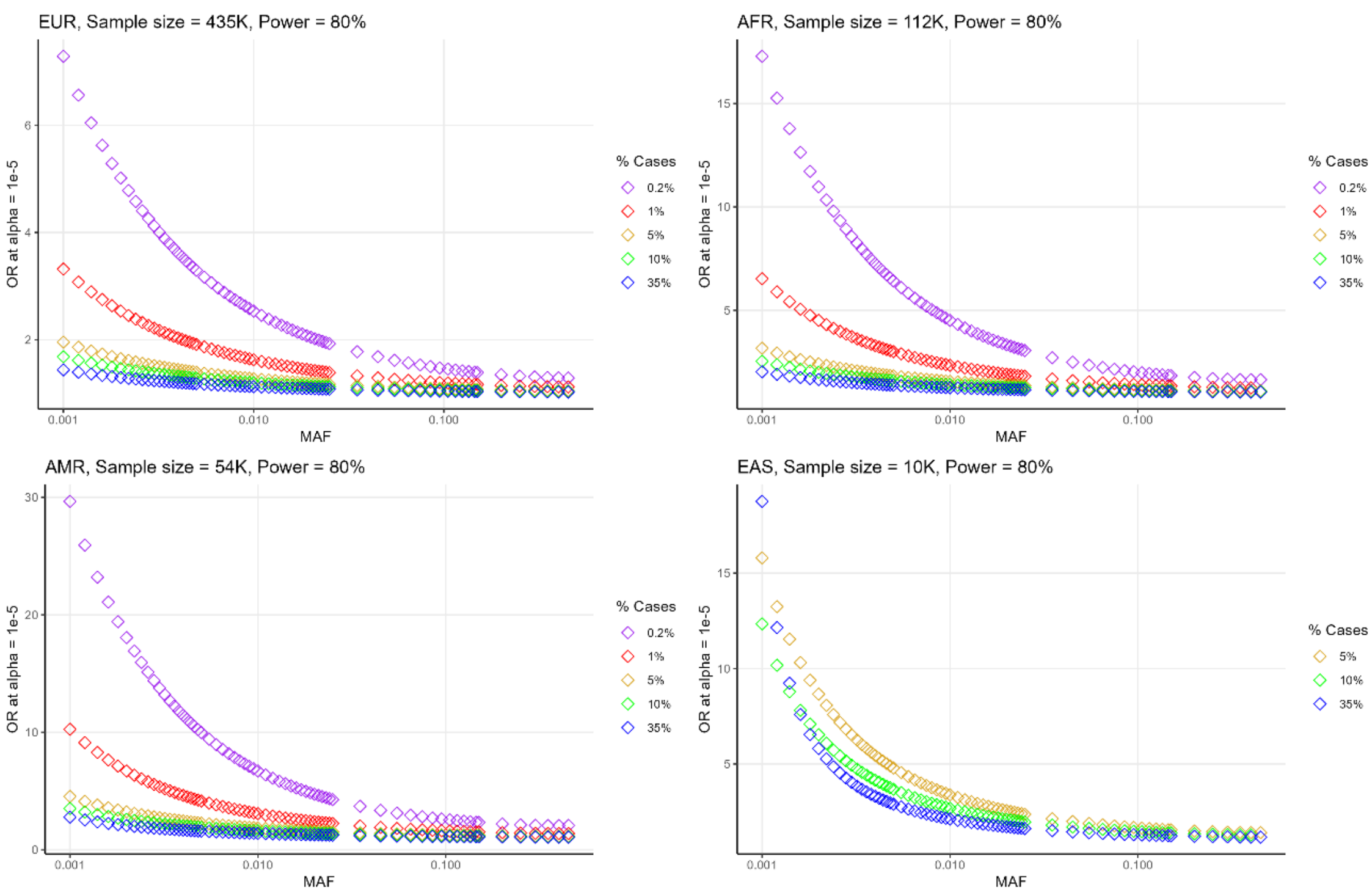

Given the fixed sample sizes in each ancestry group, with 80% power, we calculated the required sample sizes to achieve 80% power across the minor allele frequency (MAF) spectrum and across four ancestry groups.

Extended PheWAS

For all mtSNVs with significant associations in any ancestry and subgroup in the primary analysis, PheWAS analysis was performed in the same ancestry/subgroup using genotype-phenotype data from MVP (Supplementary Data 12). Phenotypes were defined by International Classification of Diseases (ICD) codes mapped to clinically relevant groups using published PheWAS methods, excluding those included in the primary analysis. The extended PheWAS results with one or more statistically significant PheCodes are shown in Supplementary Figures 9 to 11, with significant PheCode associations labeled, and source data are provided in the Source Data file.

KESER network analysis

We conducted a KESER network analysis to help interpret the identified association between mtDNA and PheCodes (Supplementary Data 5). Given a specific PheCode we identified in Table 2, Figure 2, and Figure 3, we found the neighborhood terms related to the target term, including PheCodes/ICD (International Classification of Disease) codes[1,2] for disease conditions, LOINC (Logical Observation Identifiers Names and Codes)[3] for laboratory tests, CPT (Current Procedural Terminology)[4], RxNorm for medications[5]. The neighborhood in a network trained using data from VA Corporate Data Warehouse (CDW) and Mass General Brigham (MGB) healthcare centers represents relatedness (i.e., co-occurrence) among diseases, procedures, medications, and laboratory tests. A similarity metric (cosine similarity) was also output. For each phecode, we report the top 5 unique terms with highest similarity.

Sensitivity analysis for hypothyroidism

We modified the outcome for hypothyroidism to exclude patients with certain PheCode conditions related to thyroid disease (e.g. 193-thyroid cancer), shown in Supplementary Data 9A. We then performed association tests in EUR ancestry in MVP.

To evaluate potential age-dependent effects of the reported mtSNVs on hypothyroidism, as well as potential confounding conditions, we also performed age tertile stratified analysis on the modified hypothyroidism outcome (Supplementary Data 9B). Given that the prevalence of hypothyroidism in EUR females was double that of EUR males, we separately report the sex-stratified mtSNV association analysis results in EUR ancestry (Supplementary Data 10).

MtDNA and thyroid-stimulating hormone (TSH) in UKBB

To further elucidate the relationship between reported mtSNV and hypothyroidism, we performed an additional analysis between MT226, MT10398, MT13708, MT14798, and TSH hormone in UKBB. We followed the procedure documented in a recent GWAS to capture TSH measurements reported in the primary care data available in up to 230,000 individuals in UK Biobank[6]. We utilized codes from Read version 2 and Read version 3 (Clinical Terms Version 3 or "CTV3"). An individual's first non-missing TSH measurement was extracted. This minimized the effect of thyroid function-altering medications as an individual is unlikely to have received them before their first thyroid function test. Where an individual's first non-missing TSH measurement was 0, they were excluded from the analysis unless the individual also had a code in their primary care data for hyperthyroidism, where a TSH measurement of 0 may be clinically feasible. We excluded individuals with a TSH measurement <0.4 or >4.0 mU/L as has been done previously [7]. We conducted the analysis in the cohort where hypothyroidism and other thyroid-related diseases were excluded and reported association results in the population with no hypothyroidism cases (Supplementary Data 11).

MT1555 and heart failure in MVP

We took advantage of the well-phenotyped heart failure (HF) traits and HF subtypes, namely HF with reduced ejection fraction and HF with preserved ejection fraction (HFrEF and HFpEF), in MVP patients to evaluate the association of MT1555 in female veterans with EUR ancestry[8]. In summary, HF was identified as those with an International Classification of Diseases (ICD)-9 code of 428.x or ICD-10 code of I50.x and an echocardiogram performed within 6 months of diagnosis. A comprehensive natural language processing (NLP) approach was also adopted to classify HF into HFrEF and HFpEF. This NLP tool was developed and validated in the national VA database to extract left ventricular ejection fractions (LVEF) values from echocardiogram reports, including nuclear medicine reports, cardiac catheterization reports, history and physical examination notes, and progress notes[9].

Supplementary Data 6C shows that all-cause HF and its subtypes have a higher concordance with CHF, NOS (PheCode 428.1) than with carditis (PheCode 420). Among patients diagnosed with all-cause HF, 67% also had the CHF, NOS PheCode (428.1). This percentage is comparable for HFrEF and HFpEF subtypes. As presented in Supplementary Data 6B, the estimated odds ratios (OR) for the HF phenotype and its subtypes were evaluated. We discovered an association between MT1555G and HFrEF with an OR (95%) of 5.75 (1.93 - 17.1) and a p-value of 1.7E-3.

References

1. Organization, W.H. International classification of diseases:[9th] ninth revision, basic tabulation list with alphabetic index. (1978).
2. Organization, W.H. International statistical classification of diseases and related health problems. vol. 1. (2004).
3. McDonald, C.J. *et al.* LOINC, a universal standard for identifying laboratory observations: a 5-year update. *Clin Chem* **49**, 624-33 (2003).
4. Smith, G.I. & Endicott, M. CPT update 2011. *J AHIMA* **82**, 50-1; quiz 52 (2011).
5. Nelson, S.J., Zeng, K., Kilbourne, J., Powell, T. & Moore, R. Normalized names for clinical drugs: RxNorm at 6 years. *J Am Med Inform Assoc* **18**, 441-8 (2011).
6. Williams, A.T. *et al.* Genome-wide association study of thyroid-stimulating hormone highlights new genes, pathways and associations with thyroid disease. *Nat Commun* **14**, 6713 (2023).
7. Teumer, A. *et al.* Genome-wide analyses identify a role for SLC17A4 and AADAT in thyroid hormone regulation. *Nat Commun* **9**, 4455 (2018).
8. Joseph, J. *et al.* Genetic architecture of heart failure with preserved versus reduced ejection fraction. *Nat Commun* **13**, 7753 (2022).
9. Patterson, O.V. *et al.* Unlocking echocardiogram measurements for heart disease research through natural language processing. *BMC Cardiovasc Disord* **17**, 151 (2017).

VA Million Veteran Program: Core Acknowledgement for Publications

October 2025

MVP Program Office

- Sumitra Muralidhar, Ph.D., Program Director

  US Department of Veterans Affairs, 810 Vermont Avenue NW, Washington, DC 20420
- Jennifer Moser, Ph.D., Associate Director, Scientific Programs

  US Department of Veterans Affairs, 810 Vermont Avenue NW, Washington, DC 20420
- Jennifer E. Deen, B.S., Associate Director, Cohort & Public Relations

  US Department of Veterans Affairs, 810 Vermont Avenue NW, Washington, DC 20420

MVP Steering Committee

- Co-Chair: Philip S. Tsao, Ph.D.

  VA Palo Alto Health Care System, 3801 Miranda Avenue, Palo Alto, CA 94304
- Co-Chair: Sumitra Muralidhar, Ph.D.

  US Department of Veterans Affairs, 810 Vermont Avenue NW, Washington, DC 20420
- J. Michael Gaziano, M.D., M.P.H.

  VA Boston Healthcare System, 150 S. Huntington Avenue, Boston, MA 02130
- Adriana Hung, M.D., M.P.H.,

  VA Tennessee Valley Healthcare System, 1310 24th Avenue, South Nashville, TN 37212
- Dave Oslin, M.D.

  Philadelphia VA Medical Center, 3900 Woodland Avenue, Philadelphia, PA 19104
- Deepak Voora, M.D.

  Durham VA Medical Center, 508 Fulton Street, Durham, NC 27705

MVP Co-Principal Investigators

- J. Michael Gaziano, M.D., M.P.H.

  VA Boston Healthcare System, 150 S. Huntington Avenue, Boston, MA 02130
- Philip S. Tsao, Ph.D.

  VA Palo Alto Health Care System, 3801 Miranda Avenue, Palo Alto, CA 94304

MVP Core Operations

- Jessica V. Brewer, M.P.H., Director, MVP Cohort Operations

  VA Boston Healthcare System, 150 S. Huntington Avenue, Boston, MA 02130
- Mary T. Brophy M.D., M.P.H., Director, VA Central Biorepository

  VA Boston Healthcare System, 150 S. Huntington Avenue, Boston, MA 02130

- Kelly Cho, M.P.H, Ph.D., Director, MVP Phenomics
  VA Boston Healthcare System, 150 S. Huntington Avenue, Boston, MA 02130
- Lori Churby, B.S., Director, MVP Regulatory Affairs
  VA Palo Alto Health Care System, 3801 Miranda Avenue, Palo Alto, CA 94304
- Jacob T. Kean, Ph.D., Acting Director, VA Informatics and Computing Infrastructure (VINCI)
  VA Salt Lake City Health Care System, 500 Foothill Drive, Salt Lake City, UT 84148
- Saiju Pyarajan Ph.D., Director, Data and Computational Sciences
  VA Boston Healthcare System, 150 S. Huntington Avenue, Boston, MA 02130
- Robert Ringer, Pharm.D., Director, VA Albuquerque Central Biorepository
  New Mexico VA Health Care System, 1501 San Pedro Drive SE, Albuquerque, NM 87108
- Luis E. Selva, Ph.D., Director, MVP Biorepository Coordination
  VA Boston Healthcare System, 150 S. Huntington Avenue, Boston, MA 02130
- Shahpoor (Alex) Shayan, M.S., Director, MVP PRE Informatics
  VA Boston Healthcare System, 150 S. Huntington Avenue, Boston, MA 02130
- Brady Stephens, M.S., Principal Investigator, MVP Information Center
  Canandaigua VA Medical Center, 400 Fort Hill Avenue, Canandaigua, NY 14424
- Stacey B. Whitbourne, Ph.D., Director, MVP Cohort Development and Management
  VA Boston Healthcare System, 150 S. Huntington Avenue, Boston, MA 02130
