# Supplementary material for: A large-scale multi-ancestry mitochondrial variant association analysis for cardiometabolic traits
Source: Nat Commun. 2026 Jul 4;17:8319. doi: 10.1038/s41467-026-74939-4 (PMC13469969; doi:10.1038/s41467-026-74939-4)
Supplement: Supplementary file 2 [file 41467_2026_74939_MOESM2_ESM.pdf]

Description of Additional Supplementary Files

Supplementary Data 1. Case numbers and proportions of the binary Traits (defined by PheCode) in four ancestry groups.

Supplementary Data 2. MtSNV allele frequence distribution across ancestries.

Supplementary Data 3. MtSNV Annotation.

Supplementary Data 4. Genomic Inflation Factor.

Supplementary Data 5. Identification of Co-occurring PheCodes with mtDNA-Associated PheCodes via KESER.

Supplementary Data 6. MT1555, Heart Failure (HF), and HF Subtypes in EUR females.

Supplementary Data 7. MtSNVs included in mtGene analysis.

Supplementary Data 8. Conditional analysis of the significant mtGenes, controlling for significant mtSNVs within that gene.

Supplementary Data 9. Hypothyroidism sensitivity analyses.

Supplementary Data 10. Sex Stratified MtSNVs' Results for Hypothyroidism (PheCode, 244.4) in EUR.

Supplementary Data 11. MtSNV associations with TSH in UKBB.

Supplementary Data 12. MtSNVs with significant associations in any ancestry and subgroup in the primary analysis, used for extended PheWAS analysis.

Supplementary Data 13. Full association results for all mtSNVs-trait pairs, ancestries, and subgroups.
