# Supplementary material for: A large-scale multi-ancestry mitochondrial variant association analysis for cardiometabolic traits
Source: Nat Commun. 2026 Jul 4;17:8319. doi: 10.1038/s41467-026-74939-4 (PMC13469969; doi:10.1038/s41467-026-74939-4)
Supplement: Supplementary file 3 [file 41467_2026_74939_MOESM4_ESM.pdf]

nature portfolio

Corresponding author(s): Jin J Zhou

Last updated by author(s): May 27, 2026

Reporting Summary

Nature Portfolio wishes to improve the reproducibility of the work that we publish. This form provides structure for consistency and transparency in reporting. For further information on Nature Portfolio policies, see our Editorial Policies and the Editorial Policy Checklist.

Statistics

For all statistical analyses, confirm that the following items are present in the figure legend, table legend, main text, or Methods section.

n/a	Confirmed	
☐	☒	The exact sample size (*n*) for each experimental group/condition, given as a discrete number and unit of measurement
☐	☒	A statement on whether measurements were taken from distinct samples or whether the same sample was measured repeatedly
☐	☒	The statistical test(s) used AND whether they are one- or two-sided<br>*Only common tests should be described solely by name; describe more complex techniques in the Methods section.*
☐	☒	A description of all covariates tested
☐	☒	A description of any assumptions or corrections, such as tests of normality and adjustment for multiple comparisons
☐	☒	A full description of the statistical parameters including central tendency (e.g. means) or other basic estimates (e.g. regression coefficient) AND variation (e.g. standard deviation) or associated estimates of uncertainty (e.g. confidence intervals)
☐	☒	For null hypothesis testing, the test statistic (e.g. *F*, *t*, *r*) with confidence intervals, effect sizes, degrees of freedom and *P* value noted<br>*Give P values as exact values whenever suitable.*
☒	☐	For Bayesian analysis, information on the choice of priors and Markov chain Monte Carlo settings
☒	☐	For hierarchical and complex designs, identification of the appropriate level for tests and full reporting of outcomes
☐	☒	Estimates of effect sizes (e.g. Cohen's *d*, Pearson's *r*), indicating how they were calculated

Our web collection on statistics for biologists contains articles on many of the points above.

Software and code

Policy information about availability of computer code

Data collection	No software was used to collect the data for this manuscript.
Data analysis	Data analyses were performed using R version 4.22 and plink 2.0. Code for data cleaning and analysis is available at https://github.com/jinjinzhou/mtDNA/.

For manuscripts utilizing custom algorithms or software that are central to the research but not yet described in published literature, software must be made available to editors and reviewers. We strongly encourage code deposition in a community repository (e.g. GitHub). See the Nature Portfolio guidelines for submitting code & software for further information.

Data

Policy information about availability of data

All manuscripts must include a data availability statement. This statement should provide the following information, where applicable:

- Accession codes, unique identifiers, or web links for publicly available datasets
- A description of any restrictions on data availability
- For clinical datasets or third party data, please ensure that the statement adheres to our policy

The summary statistics generated in this study from the Million Veteran Program (MVP) are provided in the Supplementary Data and Source Data files. The summary statistics from MVP are available via the dbGap study accession number phs001672 (Veterans Administration MVP Summary Results from Omics Studies). Source data are provided with this paper. Individual-level MVP data are protected and not publicly available due to participant privacy restrictions.

Research involving human participants, their data, or biological material

Policy information about studies with human participants or human data. See also policy information about sex, gender (identity/presentation), and sexual orientation and race, ethnicity and racism.

Reporting on sex and gender	The study utilized both genetically inferred sex and self-reported sex/gender obtained from survey data and/or electronic health records. Analyses were performed both stratified by sex and within both sexes combined. In both cases, participants were limited to those whose self-reported sex/gender matched their genetic sex. The majority of participants (557,036) were male, while 54,344 participants were female.
Reporting on race, ethnicity, or other socially relevant groupings	We did not utilize socially constructed race/ethnicity in this manuscript. We did utilize genetically inferred ancestry (see below). All analyses included 15 genetic principal components as potential confounding variables.
Population characteristics	Most participants were male (see above). The most common genetically inferred ancestry (GIA) was European (434,891), followed by African (111,840), Admixed American (54,258), and East Asian (10,381). The participants' mean age was 57 years, ranging from 20 to 100 years. 196,822 participants were classified as having type 2 diabetes, while 367,218 participants were determined not to have diabetes (any type).
Recruitment	The Million Veteran Program (MVP) is a large cohort of U.S. Veterans recruited from 63 participating Department of Veterans Affairs (VA) medical facilities. Recruitment started in 2011, and all Veterans were eligible for participation. Details of the recruitment, consenting and design of the study were reported previously.
Ethics oversight	VA Central Institutional Review Board (IRB)

Note that full information on the approval of the study protocol must also be provided in the manuscript.

Field-specific reporting

Please select the one below that is the best fit for your research. If you are not sure, read the appropriate sections before making your selection.

☒ Life sciences ☐ Behavioural & social sciences ☐ Ecological, evolutionary & environmental sciences

For a reference copy of the document with all sections, see nature.com/documents/nr-reporting-summary-flat.pdf

Life sciences study design

All studies must disclose on these points even when the disclosure is negative.

Sample size	After applying relevant exclusions, the total sample size was 611,370. Sample size was not predetermined; all available participants meeting study criteria were included. Power and sample size considerations are detailed in Supplementary materials.
Data exclusions	Participants for whom complete data was not available were excluded from the analyses. Complete data consisted of age, self-reported sex, genetically inferred sex, genetically inferred ancestry, and genotype / phenotype data for the corresponding analysis (PheCodes, biomarkers). Participants with ambiguous genetic sex, discordant genetic vs. self-reported sex, or a low call rate for mitochondrial SNVs (<98%) were excluded. Data were filtered to unrelated participants of European, African, Admixed American, or East Asian ancestry.
Replication	We attempted to replicate mtSNV-trait associations found in patients of European ancestry using UK Biobank (UKBB) data. Reproducibility was limited by differing phenotype availability and variation in the SNVs included on the genotyping chips utilized by UKBB and MVP. Of the associations that were able to be assessed in UKBB, 4/4 were replicated in the overall population, 1/1 in the Type 2 Diabetes (T2D) subgroup, and 0/4 in the female subgroup. No attempt was made to replicate associations found in patients of non-European ancestry.
Randomization	N/A; observational design where exposures are genetic variations. To control for confounding, all models adjusted for age, sex, and 15 genetic principal components.
Blinding	N/A; observational design.

Reporting for specific materials, systems and methods

We require information from authors about some types of materials, experimental systems and methods used in many studies. Here, indicate whether each material, system or method listed is relevant to your study. If you are not sure if a list item applies to your research, read the appropriate section before selecting a response.

Materials & experimental systems

n/a	Involved in the study
☒	☐ Antibodies
☒	☐ Eukaryotic cell lines
☒	☐ Palaeontology and archaeology
☒	☐ Animals and other organisms
☒	☐ Clinical data
☒	☐ Dual use research of concern
☒	☐ Plants

Methods

n/a	Involved in the study
☒	☐ ChIP-seq
☒	☐ Flow cytometry
☒	☐ MRI-based neuroimaging

Plants

Seed stocks	*Report on the source of all seed stocks or other plant material used. If applicable, state the seed stock centre and catalogue number. If plant specimens were collected from the field, describe the collection location, date and sampling procedures.*
Novel plant genotypes	*Describe the methods by which all novel plant genotypes were produced. This includes those generated by transgenic approaches, gene editing, chemical/radiation-based mutagenesis and hybridization. For transgenic lines, describe the transformation method, the number of independent lines analyzed and the generation upon which experiments were performed. For gene-edited lines, describe the editor used, the endogenous sequence targeted for editing, the targeting guide RNA sequence (if applicable) and how the editor was applied.*
Authentication	*Describe any authentication procedures for each seed stock used or novel genotype generated. Describe any experiments used to assess the effect of a mutation and, where applicable, how potential secondary effects (e.g. second site T-DNA insertions, mosiacism, off-target gene editing) were examined.*
