# Supplementary material for: A large-scale multi-ancestry mitochondrial variant association analysis for cardiometabolic traits
Source: Nat Commun. 2026 Jul 4;17:8319. doi: 10.1038/s41467-026-74939-4 (PMC13469969; doi:10.1038/s41467-026-74939-4)
Supplement: Supplementary file 4 [file 41467_2026_74939_MOESM5_ESM.pdf]

nature portfolio

Peer Review File

A large-scale multi-ancestry mitochondrial variant association analysis for cardiometabolic traits

Corresponding Author: Dr Jin Zhou

This file contains all reviewer reports in order by version, followed by all author rebuttals in order by version.

Version 0:

Reviewer comments:

Reviewer #1

(Remarks to the Author)
The authors have conducted an extensive association study using data from the Million Veteran Program (MVP), involving 650,000 participants. This study represents the largest examination of mitochondrial DNA (mtDNA) variations and cardiometabolic traits across multiple ancestries, including European (EUR), African (AFR), Admixed American (AMR), and East Asian (EAS). The authors implemented a comprehensive mtDNA quality control protocol for 248 genotyped loci and identified 7 EUR-specific and 2 AFR-specific mitochondrial single nucleotide polymorphisms (mtSNPs), with additional associations stratified by type 2 diabetes and sex across ancestries. Their findings emphasize the significant role of mtDNA in understanding cardiometabolic diseases. However, there are several points that need clarification and improvement:
1. Mitochondrial Variants Origin: It is unclear whether the mitochondrial variants discussed are exclusively present in the mitochondria or if some have been integrated into the nuclear genome. Clarification on this point is essential for accurate interpretation.
2. Variant Identification: The methodology used to obtain the 248 mitochondrial variants for association testing is not well explained. Additional details on how these variants were selected would enhance the transparency of the study.
3. Supplementary Figures Quality: The quality of figures in the supplementary material is subpar. The X and Y axes are not properly labeled, and the absence of legends makes the figures difficult to interpret. Improving the resolution and labeling of these figures is necessary.
4. Concordance of Allele Frequencies: The authors observed lower concordance of allele frequencies for ancestries other than Europeans. A discussion on why this might be the case is warranted.
5. Table 2 and Ancestry Limitations: In Table 2, significant mtSNV associations with cardiometabolic phenotypes are listed across various ancestry groups. However, many associations are observed significant only in Europeans, potentially due to the limited sample sizes of other ancestries. This limitation should be clearly stated in the manuscript.
6. Replication in UK Biobank (UKBB): The replication of MVP EUR mtSNV associations in the UKBB dataset raises several issues. Some SNPs were not genotyped in UKBB, and for those SNPs, association status should be checked for SNPs in strong linkage disequilibrium (LD) as proxies for replication. Additionally, there is a disparity in the alternate allele frequency and odds ratio of variant 8869/A/G under the T2D category between MVP and UKBB. The authors should provide a rationale for this discrepancy.
7. Stratification Analysis in UKBB: It is unclear if the UKBB data were analyzed using the same stratifications, specifically for females and the T2D category, before replication checks. This should be addressed to ensure consistency.
8. Direct Stratification for Cardiac Disease Phenotypes: The authors aimed to identify mitochondrial variants associated with cardiometabolic traits by stratifying samples for type 2 diabetes status and sex across ancestries. However, they did not stratify samples for clinical cardiac disease phenotypes, such as coronary atherosclerosis/congestive heart failure/ischemic heart disease/Myocardial infarction, which also have sufficient sample sizes across ancestries. The inclusion of this analysis is highly recommended to identify more relevant mitochondrial variants linked to cardiac diseases.
9. Molecular Relevance of Genetic Associations: The manuscript would benefit from exploring the molecular relevance of the identified genetic associations in mitochondria. Specifically, the impact of these variants on gene expression in relevant tissues and their potential role as expression quantitative trait loci (eQTLs) should be examined. Including examples of biological relevance would significantly strengthen the discussion.
10. Haplogroup Information: The manuscript does not describe what is haplogroup J and subgroup J1c, which should be included.
11. Reference: 1. From line 138 to 145, authors refer to Supp table 11. However, in Supp table 11, there are no descriptions of variants mentioned in the text from line 138 to 145, which makes interpretation difficult without seeing the result.

2. From line 147-148, Table 2 should be the referred table. However, it is given table 1 in text.
3. Line 149-150: Text: “this association with MT462C>T (MAF = 0.36%) persisted in both the T2D and 150 male-only subgroups within the AFR cohort (Figure 2 and Figure 3)”. However, the variant MT462C>T cannot be observed in Figure 2 and 3.
4. Line156-157: Supplementary figure 8 should be the reference instead of supplementary figure 9.
5. Line 237-238: Supplementary figure 13 is the reference instead of Supplementary figure 12.
12. Rare Variant Definition: The rationale behind defining rare variants as those with a minor allele frequency (MAF) of <2% (Figure 1) needs to be explained.
13. Study Power: The manuscript should provide information on the study's power to detect both common and rare variants across the four ancestries.
14. Genotyping Chip Selection: The authors chose a genotyping chip that includes mostly rare variants. However, the manuscript does not clarify why common mitochondrial variants, which may also be relevant to cardiometabolic traits, were not included. The reasoning behind this decision should be discussed.
15. Adjusted p-values in Table 2: The methodology for calculating adjusted p-values in Table 2 is unclear. The results with adjusted p-values < 0.05 were highlighted, but the manuscript also mentions controlling the significance level at a false discovery rate (FDR) of 5%, which will correspond to a p-value of 2.02 x 10-4 (0.05/number of tested variants = 0.05/248). This discrepancy needs to be addressed.

Reviewer #2

(Remarks to the Author)
In this extensive analysis of the MVP cohort, the authors assessed the contributions of mitochondrial SNPs to cardiometabolic traits in several ethnicities. The data analysis has been conducted impeccably.

Several issues need to be addressed

1. This paper is essentially a replication of a landmark paper published by UK Biobank investigators in 2021 that was published in Nature Genetics (REFERENCE #4 IN in this paper). A deeper discussion of the differences in the findings between this study and the previous one is warranted.
2. No attempt is made to try to explain the potential effects of the variants on the phenotypes that are affected by them. There are many ways that these SNPs can modulate the biological processes involved in mitochondrial function, from effects on the 13 large genes encoded by the mtDNA to multiple other processes.
3. The references are remarkably minimal. The authors should quote the multiple previous studies that conducted similar analyses prior to theirs and the multiple insights gained, from the interface between mito-genetics and disease in general to specific SNPs associated with certain phenotypes, including both SNPs that the authors did not identify in the current study but were recently published, to papers who have published on the very SNPs identified in the current study as being pathogenic, yet were not quoted in this manuscript.
4. For example, the MT228G>A variant the authors identified was previously published to be associated with early cataract development in Hispanics. Similarly, the MT13708G>A variant the authors identified was previously published to be associated with breast cancer risk.

Version 1:

Reviewer comments:

Reviewer #1

(Remarks to the Author)
All the comments have been addressed by authors. I have no more comments.

Reviewer #2

(Remarks to the Author)
The manuscript has been revised in a mostly appropriate manner

Open Access This Peer Review File is licensed under a Creative Commons Attribution 4.0 International License, which permits use, sharing, adaptation, distribution and reproduction in any medium or format, as long as you give appropriate credit to the original author(s) and the source, provide a link to the Creative Commons license, and indicate if changes were made.
In cases where reviewers are anonymous, credit should be given to 'Anonymous Referee' and the source.
The images or other third party material in this Peer Review File are included in the article's Creative Commons license, unless indicated otherwise in a credit line to the material. If material is not included in the article's Creative Commons license and your intended use is not permitted by statutory regulation or exceeds the permitted use, you will need to obtain permission directly from the copyright holder.
To view a copy of this license, visit https://creativecommons.org/licenses/by/4.0/

Response to "A large-scale multi-ancestry mitochondrial variant association analysis for cardiometabolic traits"

We thank the editor and reviewers for their careful review, which has improved our manuscript. We have addressed all concerns in the current revision. Below, the reviewers' comments are in *italic*s, and our responses appear in plain type.

Global updates during revision

During revision, we added a small number of **conservative downstream filters and reporting updates** (building on the same raw-data QC). We re-ran affected analyses and updated the corresponding tables, figures, and text:

- Strengthened QC filters: applied >98% sample and variant call-rate thresholds and removed individuals with ambiguous or discordant genetic sex to ensure consistent stratified analyses.
- Updated gnomAD database to v4.1, refreshed reference allele frequencies, and updated the association pipeline to support phenotype-specific allele-frequency outputs.
- Removed congenital anomaly phenotypes; updated to SNP-specific case counts (accounting for missing genotypes); removed results that no longer met inclusion thresholds after these fixes.
- Corrected gene-based annotation: fixed transcript mapping so a previously duplicated SNP-to-gene assignment is now correctly assigned to a single mitochondrial gene.

Editors' Comments, if provided:

(There are no comments.)

REVIEWER COMMENTS:

Reviewer #1 (Remarks to the Author):

The authors have conducted an extensive association study using data from the Million Veteran Program (MVP), involving 650,000 participants. This study represents the largest examination of mitochondrial DNA

(mtDNA) variations and cardiometabolic traits across multiple ancestries, including European (EUR), African (AFR), Admixed American (AMR), and East Asian (EAS). The authors implemented a comprehensive mtDNA quality control protocol for 248 genotyped loci and identified 7 EUR-specific and 2 AFR-specific mitochondrial single nucleotide polymorphisms (mtSNPs), with additional associations stratified by type 2 diabetes and sex across ancestries. Their findings emphasize the significant role of mtDNA in understanding cardiometabolic diseases. However, there are several points that need clarification and improvement:

1. *Mitochondrial Variants Origin: It is unclear whether the mitochondrial variants discussed are exclusively present in the mitochondria or if some have been integrated into the nuclear genome. Clarification on this point is essential for accurate interpretation.*

   Response: Over long evolutionary time scales, copies of mitochondrial DNA (mtDNA) have been transferred into the nuclear genome (nuMTs). Variant sites in nuMTS can be mistaken for very low-level heteroplasmy in mtDNA, but because of the high number of mtDNA copies per nuclear copy (>100 in blood samples), nuMTs do not affect the assessment of fixed mtDNA variants, which is what we are analyzing in this paper. We have edited the text on page 16 ("**Genotyping and quality control" section)** to clarify that the analysis of nuclear mitochondrial variants/genes was not within the scope of this paper.

2. *Variant Identification: The methodology used to obtain the 248 mitochondrial variants for association testing is not well-explained. Additional details on how these variants were selected would enhance the transparency of the study.*

   Response: We have updated the "**Genotyping and Quality Control"** section in both the main text and the Supplementary Methods to include additional details. In the main text (page 16), we revised the description to:

   > "Only a small fraction of the mitochondrial variants present on the chip reach appreciable frequency in the population. Most mitochondrial variants were selected for inclusion based on literature reporting variants of strong effect in rare mitochondrial diseases. Thus, there is a significant bias toward low–allele-frequency variants."

3. *Supplementary Figures Quality: The quality of figures in the supplementary material is subpar. The X and Y axes are not properly labeled, and the absence of legends makes the figures difficult to interpret. Improving the resolution and labeling of these figures is necessary.*

   Response: We have updated these figures to include the proper labels and substantially improved the presentation. See Supplementary Figures 2-6 in the supplementary materials, pages 3-7.

4. *Concordance of Allele Frequencies: The authors observed lower concordance of allele frequencies for ancestries other than Europeans. A discussion on why this might be the case is warranted.*

   Response: We appreciate the reviewer's comments. In this revision, we updated our analysis to use the latest gnomAD version (v4.1 rather than v3.1). The allele frequencies we report for non-European populations exhibit greater random variation due to smaller sample sizes. Nevertheless, the slope of the relationship between our allele frequencies and those from gnomAD remains close to 1 across all ancestries, indicating strong agreement. We have revised the manuscript on page 5 (**"Characteristics of study participants and mtDNA variants"** section) to reflect this point.

5. *Table 2 and Ancestry Limitations: In Table 2, significant mtSNV associations with cardiometabolic phenotypes are listed across various ancestry groups. However, many associations are observed significant only in Europeans, potentially due to the limited sample sizes of other ancestries. This limitation should be clearly stated in the manuscript.*

   Response: We agree. The observation is largely explained by smaller sample sizes in non-European groups and by lower allele frequencies of these variants in those populations (notably, Europeans have the highest allele frequency for 7 of the 8 significant associations). We have revised the discussion (page 15, the 6th limitation in the "**Discussion**" section) to clearly state this limitation.

6. *Replication in UK Biobank (UKBB): The replication of MVP EUR mtSNV associations in the UKBB dataset raises several issues. Some SNPs were not genotyped in UKBB, and for those SNPs, association status should be checked for SNPs in strong linkage disequilibrium (LD) as proxies for replication. Additionally, there is a disparity in the alternate allele frequency and odds ratio of variant 8869/A/G under the T2D category between MVP and UKBB. The authors should provide a rationale for this discrepancy.*

   Response: Since mtDNA is inherited as a single, non-recombining unit, there is effectively no recombination between positions, and variants are inherited in complete haplotypes. Within a haplogroup, mtDNA variants tend to be perfectly correlated unless altered by new mutation or

heteroplasmy. Because of the high mutation rate in mtDNA, recurrent mutations at the same site are also relatively common, further reducing the utility of conventional LD-based tagging methods. In this analysis, we therefore prioritized individual mtSNV associations rather than broader haplogroup-level tests. For replication in UKBB, we restricted analyses to mtSNVs that were directly genotyped in both cohorts and did not use proxy variants.

Due to the design of the MVP genotyping chip, which is enriched for rare variants, reliable haplogroup assignment was not feasible for most individuals. In response to the reviewer's suggestion, we attempted haplogroup inference using Haplogrep 2.0. The mean haplotype quality scores ranged from 0.589 (AMR) to 0.678 (AFR), where a score of 1 reflects the highest confidence, and 0 indicates no confidence, confirming that mitochondrial haplogroup determination is not practical with this platform.

For MT8869A>G, the point estimate of the odds ratio (OR) is higher in UKBB than in MVP (33.5 vs. 2.97), but the confidence intervals overlap (UKBB: 1.88–594; MVP: 1.84–4.79). The very wide CI in UKBB likely reflects the low allele frequency and small number of carriers. In MVP, the alternative-allele frequency of 0.45% in European ancestry agrees well with the gnomAD non-Finnish European frequency (0.36%), whereas the UKBB frequency is ten-fold lower (0.04%). Although both cohorts are predominantly European, rare mitochondrial allele frequencies can vary substantially due to founder effects and population history. For example, MT8869A>G is most common in the V1 sub-haplogroup,
(*https://www.mitomap.org/haploinfo_genbank/A8869G.html*;
https://hras.yseq.net/hras.php?dna_type=mt),
which is more frequent in Scandinavian and Baltic populations than in the British Isles. These historical and geographic differences likely contribute to the observed divergence. We have clarified these points in the main manuscript (pages 14 ("limitations" of the "**Discussion**" section) and 17 "**Genotyping and quality control**" section).

7. *Stratification Analysis in UKBB: It is unclear if the UKBB data were analyzed using the same stratifications, specifically for females and the T2D category, prior to replication checks. This should be addressed to ensure consistency.*

Response: UKBB data were analyzed in subgroups (females, T2D) only for replication purposes, i.e., to identify significant associations in MVP for the same subgroup and trait-locus pair. We have added more information about the UKBB stratified analyses to the text (page 20, "**Replication in UKBB**") to clarify how they were performed.

8. *Direct Stratification for Cardiac Disease Phenotypes: The authors aimed to identify mitochondrial variants associated with cardiometabolic traits by stratifying samples for type 2 diabetes status and sex across ancestries. However, they did not stratify samples for clinical cardiac disease phenotypes, such as coronary atherosclerosis/congestive heart failure/ischemic heart disease/Myocardial infarction, which also have sufficient sample sizes across ancestries. The inclusion of this analysis is highly recommended to identify more relevant mitochondrial variants linked to cardiac diseases.*

Response: We appreciate this thoughtful comment. In the current manuscript, cardiac diseases are already analyzed as **primary outcomes**, motivated by prior reports linking mtDNA to cardiac phenotypes[1-4]. Notably, ischemic heart disease (IHD; PheCode 411, observed at frequencies ranging from 19.1% to 38.7%) is highly correlated with several other cardiac PheCodes (Table below). For example, among individuals of European ancestry with IHD, 93% also had a diagnosis of hypertension (PheCode 401). In addition, some cardiac PheCodes, such as myocardial infarction (MI; PheCode 411.2), are entirely subsumed under IHD in the PheCode hierarchy. Given this extensive overlap, it is unclear what additional information would be gained by stratifying analyses on individual cardiac PheCodes (e.g., assessing associations of mtSNVs with hypertension or heart failure in the presence versus absence of IHD), and such conditioning on cardiac outcomes could also introduce collider bias.

We stratified our analyses by diabetes status because of the well-established role of mitochondria in type 2 diabetes pathophysiology[5-10] and the distinct cardiometabolic profiles of individuals with and without T2D. Sex and T2D strata were defined *a priori* and reflected our main mechanistic hypotheses. Further stratification by specific cardiac conditions would substantially reduce power for other cardiometabolic traits (e.g., liver, endocrine, or congenital disorders) and greatly increase the number of exploratory tests, with subgroup results unlikely to differ meaningfully from the whole-cohort analyses. For these reasons, we did not perform additional cardiac-status–stratified analyses but instead report mtDNA associations with major cardiac phenotypes directly.

Phecode	Phenotype	EUR (n, %)	AFR (n, %)	AMR (n, %)	EAS (n, %)

411	Ischemic Heart Disease	169,193 (38.7%)	35,322 (31%)	14,611 (26.7%)	2,005 (19.1%)
411.4	Coronary Atherosclerosis	147,578 (33.8%)	25,897 (22.7%)	12,720 (23.2%)	1,600 (15.2%)
428	Congestive Heart Failure, Nonhypertensive	77,905 (17.7%)	19,641 (17.3%)	9,038 (16.5%)	814 (7.7%)
411.2	Myocardial Infarction	56,215 (12.8%)	11,670 (10.2%)	4,679 (8.6%)	627 (6%)

9. *Molecular Relevance of Genetic Associations: The manuscript would benefit from exploring the molecular relevance of the identified genetic associations in mitochondria. Specifically, the impact of these variants on gene expression in relevant tissues and their potential role as expression quantitative trait loci (eQTLs) should be examined. Including examples of biological relevance would significantly strengthen the discussion.*

   Response: We agree that functional context strengthens interpretation. However, current public eQTL resources do not provide a systematic framework for ***cis*-**eQTLs of mtDNA-encoded transcripts (and such analyses are highly dependent on heteroplasmy and mtDNA copy number), so a formal mtDNA-eQTL analysis is not feasible with the present data. Instead, we now expand the biological context for our lead signals in the **Discussion**.

   Most coding variants in Table 2 carry *benign/likely benign* ClinVar labels, as expected for common variants with small effects on complex traits rather than Mendelian mitochondrial disease. Nevertheless, there is relevant functional literature. For example, MT10398A>G (ND3 p.Thr114Ala) — one of our replicated hypothyroidism signals—has been studied in cybrid systems and linked to altered complex I bioenergetics, reactive oxygen species generation, and apoptosis; molecular consequences for *ND3* have also been characterized[11,12]. We also discuss how haplogroup-J–tagging variants at complex I (*ND3* 10398A>G) and complex III (*CYB* 14798T>C), together with the D-loop 228 control-region variant, provide a plausible route from mtDNA variation to altered bioenergetic coupling/redox set points and higher TSH among euthyroid participants—consistent with the observed hypothyroidism associations and our UKBB TSH analysis. We have added these points to the "**Discussion"** section (pages 12 - 14).

10. *Haplogroup Information: The manuscript does not describe what is haplogroup J and subgroup J1c, which should be included.*

Response: We appreciate the comment and have edited the text to include additional information about haplogroups J and J1c, namely, the geographic locations where they are typically found and at what frequency. See page 6, lines 164-167.

11. *Reference: 1. From line 138 to 145, authors refer to Supp table 11. However, in Supp table 11, there are no descriptions of variants mentioned in the text from line 138 to 145, which makes interpretation difficult without seeing the result.*

    Response: We appreciate the comment. Supplementary Table 11 (now Supplementary Table 5 after re-arrangement) comprises PheCode similarity metrics extracted by KESER. It does not contain SNP-based information or association results.

12. *From lines 147-148, Table 2 should be the referred table. However, it is given table 1 in text.*

    Response. We appreciate the comment and have made the necessary corrections in the manuscript (page 7).

13. *Line 149-150: Text: "this association with MT462C>T (MAF = 0.36%) persisted in both the T2D and 150 male-only subgroups within the AFR cohort (Figure 2 and Figure 3)". However, the variant MT462C>T cannot be observed in Figures 2 and 3.*

    Response. We have revised the manuscript. We have also clarified which variants are included in the interaction analyses, i.e., "For mtSNVs significantly associated with a phenotype in one subgroup, but not in the overall group for that ancestry, we analyzed and tested for statistical interactions with the relevant stratifying variable (T2D status or sex) using logistic regression". See page 19 ("**MtSNV association analyses**" section).

14. *Line 156-157: Supplementary figure 8 should be the reference instead of supplementary figure 9.*

    Response. We appreciate the comment and have made the necessary corrections in the manuscript.

15. *Line 237-238: Supplementary figure 13 is the reference instead of Supplementary figure 12.*

    Response. We appreciate the comment and have made the necessary corrections in the manuscript.

16. *Rare Variant Definition: The rationale behind defining rare variants as those with a minor allele frequency (MAF) of <2% (Figure 1) needs to be explained.*

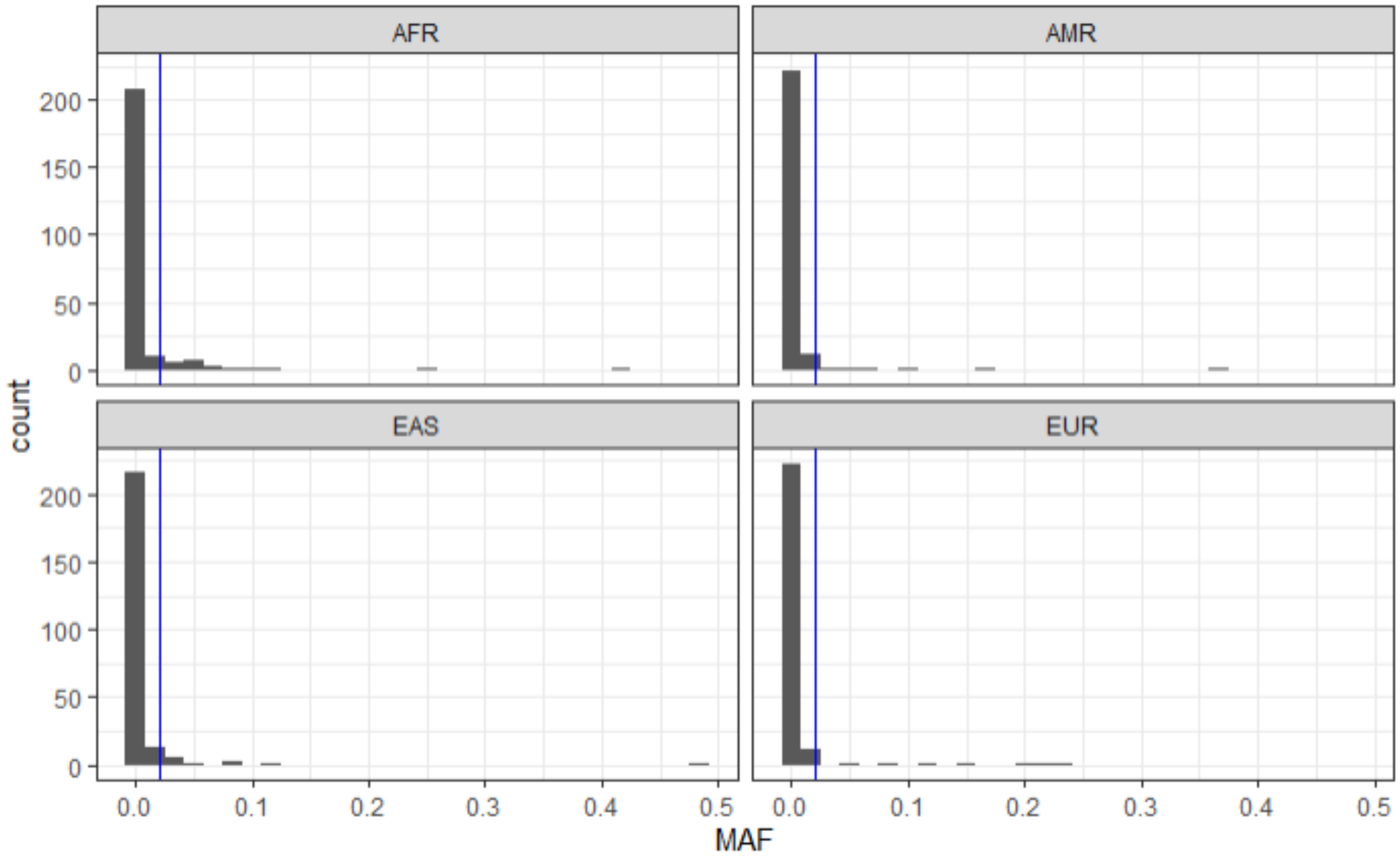

Figure 1. Minor allele frequency (MAF) distribution across ancestry groups in MVP. The blue vertical line indicates 2% MAF.

Response. We used a MAF of 2% to be consistent with the prior published atlas of mitochondrial DNA genotype-phenotype associations in the UK Biobank [13]. This threshold also allowed us to use most mtSNPs in gene-based analyses, as the majority fell below it (**Figure 1 above**). We have clarified this in the manuscript text (page 10, "**Mitochondrial gene-based rare variant burden analysis**" section).

17. *Study Power: The manuscript should provide information on the study's power to detect both common and rare variants across the four ancestries.*

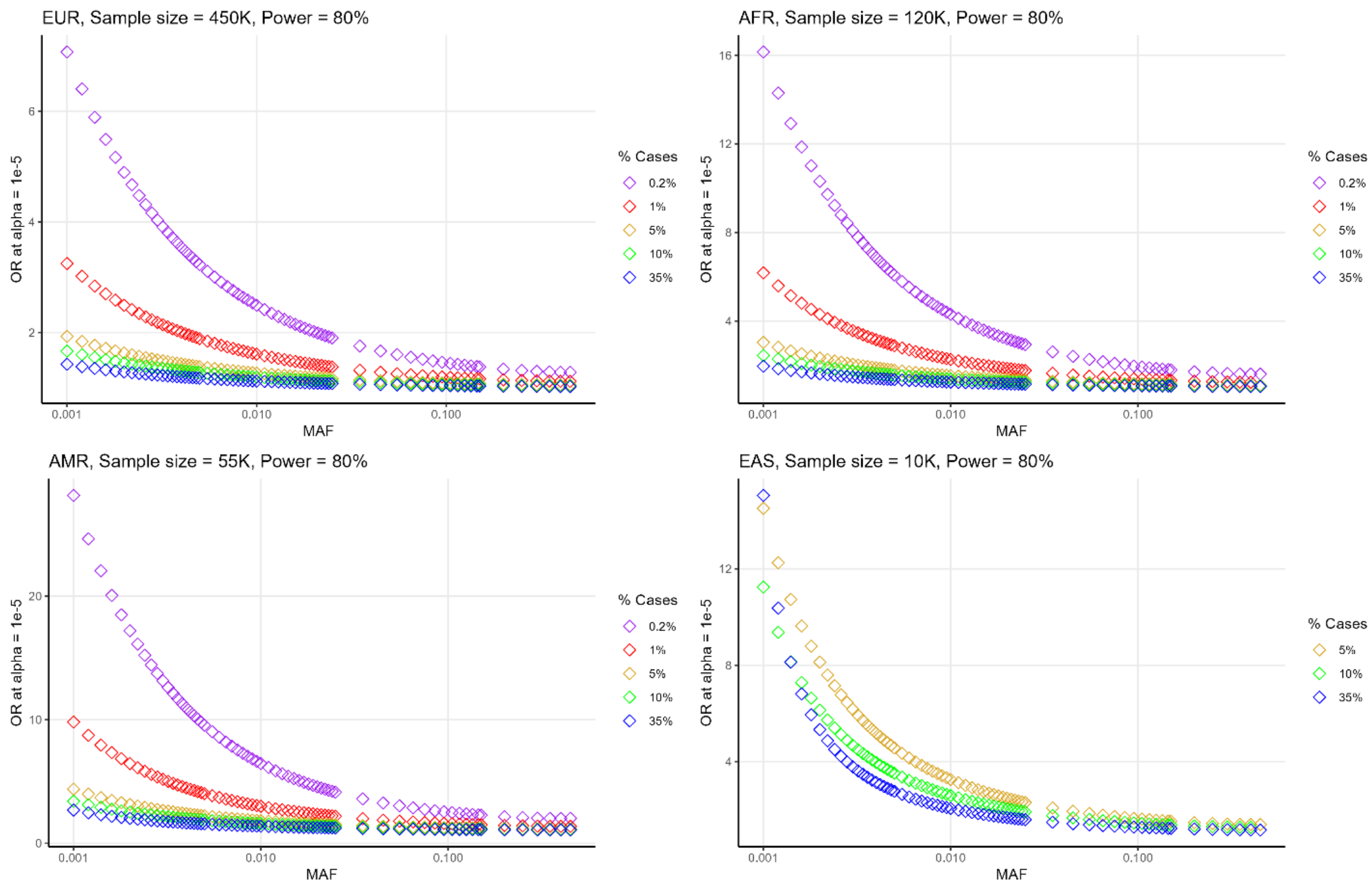

Figure 2. Given the fixed sample sizes in each ancestry group, we calculated the required sample sizes to achieve 80% power across the minor allele frequency (MAF) spectrum and across four ancestry groups.

Response: We agree that it is important to quantify power, and we have now done so by simulation for each ancestry (**Figure 2 above and Supplementary Power** section). In the European ancestry group (~435,000 participants), power curves show that, at a study-wide $\alpha = 1\times10^{-5}$, we achieve 80% power to detect: (i) a rare allele (MAF = 0.001) when the odds ratio (OR) is 1.7 for a trait with 10% prevalence, and (ii) a rare trait (1% prevalence) when the MAF is 1% and the OR is 1.6. When both the allele frequency and trait prevalence are low (MAF = 0.001 and prevalence = 1%), an OR >3.3 is required for 80% power.

As expected, ancestries with smaller sample sizes (AFR, AMR, EAS) have reduced power, particularly for low-frequency variants and rarer traits. In these groups, fewer SNP–trait pairs were tested, and the typical case proportion was higher because we required ≥500 cases per ancestry-specific analysis. Consistent with these power curves, most of the associations detected in EUR involved common variants and/or relatively frequent phenotypes. For the associations we reported, the predicted power (at $\alpha = 1\times10^{-5}$), given the observed MAF, trait frequency, and sample size, was >75% for EUR and >69% for AFR. The full simulation details and ancestry-specific power curves are now provided in the Supplementary Materials.

18. *Genotyping Chip Selection: The authors chose a genotyping chip that includes mostly rare variants. However, the manuscript does not clarify why common mitochondrial variants, which may also be relevant to cardiometabolic traits, were not included. The reasoning behind this decision should be discussed.*

    Response. The MVP 1.0 array is derived from the Applied Biosystems Axiom Biobank Genotyping Array, with MVP-specific content added to enrich for rare and functional variations relevant to translational research. As detailed previously[14], the backbone provides genome-wide coverage of common variants. At the same time, custom content emphasizes rare coding SNPs/indels, pharmacogenomic loci, eQTLs, and loss-of-function markers, including content to improve imputation in African-ancestry samples. In total, 686,682 unique bi-allelic markers were tiled, including 270 mitochondrial markers. Regarding the reviewer's question: we did not design the array. It was designed by the Million Veteran Program, and due to the prioritization of specific categories, 270 mitochondrial markers were enriched with rare variants.

    We added language clarifying this design choice in the manuscript and directed readers to the prior manuscript, see page 16 (section "**Genotyping and quality control**").

19. *Adjusted p-values in Table 2: The methodology for calculating adjusted p-values in Table 2 is unclear. The results with adjusted p-values < 0.05 were highlighted, but the manuscript also mentions controlling the significance level at a false discovery rate (FDR) of 5%, which will correspond to a p-value of 2.02 x 10-4 (0.05/number of tested variants = 0.05/248). This discrepancy needs to be addressed.*

    Response. We have clarified in the text (page 18, the "**Statistical analysis"** section) that the number of associations tested in each population varies according to case counts and allele frequencies (variants must have MAF > 0.001 and traits must have > 500 cases). Therefore, the FDR significance threshold also varies across different populations.

Reviewer #2 (Remarks to the Author):

In this extensive analysis of the MVP cohort, the authors assessed the contributions of mitochondrial SNPs to cardiometabolic traits in several ethnicities. The data analysis has been conducted impeccably.

Several issues need to be addressed:

1. *This paper is essentially a replication of a landmark paper published by UK Biobank investigators in 2021 that was published in Nature Genetics (REFERENCE #4 IN in this paper). A deeper discussion of* the differences in the findings between this study and the previous one is warranted.

   Response: While the current analysis bears many similarities to the 2021 UKBB paper, it also has important differences and strengths. Most importantly, our analysis also includes patients of non-European ancestries. Additionally, the genotyping chip used contains many variants that were not available in UKBB. Finally, UKBB EHR linkage lacks primary care data for most patients, meaning that phenotyping relies heavily on hospital records and self-report at the UKBB assessment center visit. Consequently, we report higher prevalences of many outcomes in MVP, which reduces misclassification and improves statistical power to detect associations. All of these comments were discussed throughout the manuscript.

2. *No attempt is made to try to explain the potential effects of the variants on the phenotypes that are affected by them. There are many ways that these SNPs can modulate the biological processes involved in mitochondrial function, from effects on the 13 large genes encoded by mtDNA to multiple other processes.*

   Response: We appreciate your comment and agree that an explanation of the potential effects of the variants on the phenotypes would be necessary to strengthen the manuscript. In the manuscript, we mention which SNPs are non-synonymous variants and therefore are expected to impact the resulting protein. The function of these proteins is so ubiquitous that alterations in function would not point to any specific mechanisms, such as pathways. These proteins are essential to all processes that require ATP and are active in nearly all cells.

   For example, MT10398A>G (rs2853826) is a common and one of the most extensively studied variants. As noted in Table 2, this is a non-synonymous missense mutation in *MT-ND3* that has been linked to

various human diseases[11,12,15,16]. It has also been demonstrated to have measurable differences in reactive oxygen species generation and apoptosis rate compared to other variants in cytoplasmic hybrid (cybrid) cell model systems[11]. Additionally, its molecular effects on the *MT-ND3* electron transport protein have been characterized. (see Table 7 in this reference[12]). We have updated the manuscript to discuss this, see pages 12-15.

3. *The references are remarkably minimal. The authors should quote the multiple previous studies that conducted similar analyses prior to theirs and the multiple insights gained, from the interface between mito-genetics and disease in general to specific SNPs associated with certain phenotypes, including both SNPs that the authors did not identify in the current study but were recently published, to papers who have published on the very SNPs identified in the current study as being pathogenic, yet were not quoted in this manuscript.*

   Response. We appreciate the comment. Although the journal requires the literature cited to be less than 70, we managed to add more references as suggested.

4. *For example, the MT228G>A variant the authors identified was previously published to be associated with early cataract development in Hispanics. Similarly, the MT13708G>A variant the authors identified was previously published to be associated with breast cancer risk.*

   Response. Thank you for pointing out these previously reported associations. We have now included the suggested references in our manuscript. Our study primarily focuses on cardiometabolic outcomes; therefore, in our literature review, we emphasized pathogenic effects of variants on cardiometabolic traits or other common/complex diseases. For this reason, we included the MT228 variant, given its reported association with early cataract development in the Hispanic population (see page 12).

References

1. Norheim, F. *et al.* Gene-by-Sex Interactions in Mitochondrial Functions and Cardio-Metabolic Traits. *Cell Metab* **29**, 932-949 e4 (2019).
2. Liu, C. *et al.* Association of genetic variation in the mitochondrial genome with blood pressure and metabolic traits. *Hypertension* **60**, 949-56 (2012).
3. El-Hattab, A.W. & Scaglia, F. Mitochondrial Cardiomyopathies. *Front Cardiovasc Med* **3**, 25 (2016).
4. Chow, J., Rahman, J., Achermann, J.C., Dattani, M.T. & Rahman, S. Mitochondrial disease and endocrine dysfunction. *Nat Rev Endocrinol* **13**, 92-104 (2017).
5. Szendroedi, J., Phielix, E. & Roden, M. The role of mitochondria in insulin resistance and type 2 diabetes mellitus. *Nat Rev Endocrinol* **8**, 92-103 (2011).
6. Sun, J. *et al.* African Mitochondrial DNA Haplogroup L2 Is Associated With Slower Decline of beta-cell Function and Lower Incidence of Diabetes Mellitus in Non-Hispanic, Black Women Living With Human Immunodeficiency Virus. *Clin Infect Dis* **71**, e218-e225 (2020).
7. Poulton, J. *et al.* Type 2 diabetes is associated with a common mitochondrial variant: evidence from a population-based case-control study. *Hum Mol Genet* **11**, 1581-3 (2002).
8. Nakano, S. *et al.* Mitochondrial DNA point mutation at nucleotide pair 3316 in a Japanese family with heterogeneous phenotypes of diabetes. *Endocr J* **45**, 625-30 (1998).
9. Nakagawa, Y. *et al.* A new mitochondrial DNA mutation associated with non-insulin-dependent diabetes mellitus. *Biochem Biophys Res Commun* **209**, 664-8 (1995).
10. Liou, C.W. *et al.* A common mitochondrial DNA variant and increased body mass index as associated factors for development of type 2 diabetes: Additive effects of genetic and environmental factors. *J Clin Endocrinol Metab* **92**, 235-9 (2007).
11. Liou, C.W. *et al.* Mitochondrial DNA variants as genetic risk factors for Parkinson disease. *Eur J Neurol* **23**, 1289-300 (2016).
12. Castaneda, V., Haro-Vinueza, A., Salinas, I., Caicedo, A. & Mendez, M.A. The MitoAging Project: Single nucleotide polymorphisms (SNPs) in mitochondrial genes and their association to longevity. *Mitochondrion* **66**, 13-26 (2022).
13. Yonova-Doing, E. *et al.* An atlas of mitochondrial DNA genotype-phenotype associations in the UK Biobank. *Nat Genet* **53**, 982-993 (2021).
14. Hunter-Zinck, H. *et al.* Genotyping Array Design and Data Quality Control in the Million Veteran Program. *Am J Hum Genet* **106**, 535-548 (2020).
15. Shoffner, J.M. *et al.* Mitochondrial DNA variants observed in Alzheimer disease and Parkinson disease patients. *Genomics* **17**, 171-84 (1993).
16. Juo, S.H. *et al.* A common mitochondrial polymorphism 10398A>G is associated metabolic syndrome in a Chinese population. *Mitochondrion* **10**, 294-9 (2010).
